# Embedded Microbubbles for Acoustic Manipulation of Single Cells and Microfluidic Applications

**DOI:** 10.1021/acs.analchem.1c01209

**Published:** 2021-07-06

**Authors:** Nino F. Läubli, Michael S. Gerlt, Alexander Wüthrich, Renard T. M. Lewis, Naveen Shamsudhin, Ulrike Kutay, Daniel Ahmed, Jürg Dual, Bradley J. Nelson

**Affiliations:** † Department of Mechanical and Process Engineering, ETH Zurich, Multi-Scale Robotics Lab, Institute of Robotics and Intelligent Systems, Tannenstrasse 3, 8092 Zurich, Switzerland; ‡ Molecular Neuroscience Group, Department of Chemical Engineering and Biotechnology, University of Cambridge, Philippa Fawcett Drive, CB3 0AS Cambridge, United Kingdom; § Department of Mechanical and Process Engineering, ETH Zurich, Mechanics and Experimental Dynamics, Institute of Mechanical Systems, Tannenstrasse 3, 8092 Zurich, Switzerland; ∥ Department of Biology, ETH Zurich, Institute of Biochemistry, Otto-Stern-Weg 3, 8093 Zurich, Switzerland; ⊥ Department of Mechanical and Process Engineering, ETH Zurich, Acoustic Robotics Systems Lab, Säumerstrasse 4, 8803 Rüschlikon, Switzerland

## Abstract

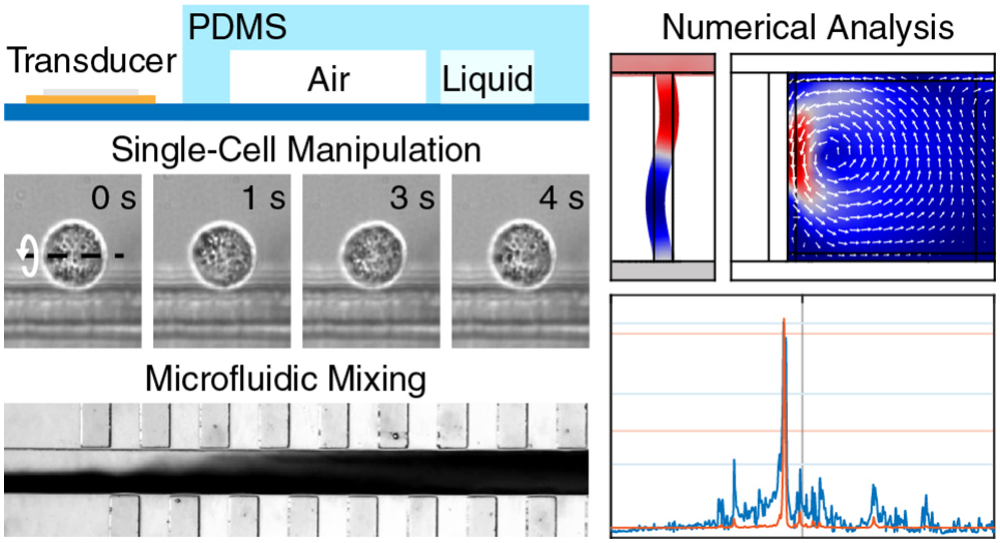

Acoustically excited microstructures have demonstrated significant potential for small-scale biomedical applications by overcoming major microfluidic limitations. Recently, the application of oscillating microbubbles has demonstrated their superiority over acoustically excited solid structures due to their enhanced acoustic streaming at low input power. However, their limited temporal stability hinders their direct applicability for industrial or clinical purposes. Here, we introduce the embedded microbubble, a novel acoustofluidic design based on the combination of solid structures (poly(dimethylsiloxane)) and microbubbles (air-filled cavity) to combine the benefits of both approaches while minimizing their drawbacks. We investigate the influence of various design parameters and geometrical features through numerical simulations and experimentally evaluate their manipulation capabilities. Finally, we demonstrate the capabilities of our design for microfluidic applications by investigating its mixing performance as well as through the controlled rotational manipulation of individual HeLa cells.

## Introduction

Precise manipulation capabilities at small scales are of increasing importance for a wide variety of research fields such as biomedicine or biology, e.g., for the investigation of morphogenesis through single-cell analysis or the detailed three-dimensional (3D) reconstruction of complex model organisms, a tool crucial for the study of extracellular features as well as the internal organs necessary for preclinical drug development.^1,2^ Furthermore, the subsequent ability to access multiple regions of a single specimen, e.g., for precise injections^3^ or mechanical characterizations,^4^ opens new pathways for microorganism-centered biomedical research by reducing noise induced through biological variation. Therefore, it is not surprising that a large number of techniques have been introduced to facilitate the controlled rotation and manipulation of small particles based on mechanical, magnetic, electrical, hydrodynamic, and optical forces.^5^ For example, optical traps are typically applied for the high-resolution manipulation of particles ranging from a few nanometers to micrometers by focusing light with high numerical aperture objectives^6,7^ and current advances demonstrated their use to drive single cell-based micromotors.^8^ The development of photosensitive substrates enabled the generation of various electrode patterns and subsequently the application of optoelectronic tweezers for single-cell trapping.^9^ Other contact-free manipulation methods make use of acoustic waves which, e.g., have been presented as being suitable to power nanorods inside living HeLa cells.^10^ Furthermore, both techniques have recently been combined into a sono-optical device to facilitate the controlled full 3D rotation of cancer cell spheroids.^11^

Unfortunately, many of these techniques rely on specific properties of the specimen, which significantly reduces their applicability for biological samples, limits them to specific sizes, or requires expensive or custom-made equipment. In contrast, the controlled particle manipulation using acoustic forces, i.e., acoustophoresis, has been widely applied due to its advantages of being label-free, contactless, and flexible in design while exposing biocompatible behavior.^12,13^

Recent publications have illustrated a large variety of microstructures suitable for the controlled manipulation of single cells and particles through acoustic forces. Solid features such as poly(dimethylsiloxane) (PDMS) or silicon sharp edges are acoustically excited *via* an external piezoelectric transducer, leading to the formation of acoustic streaming patterns in the nearby liquid.^14−19^ Unfortunately, in contrast to surface acoustic wave devices or bubble-based designs, the majority of acoustofluidic setups relying on the generation of vortices *via* solid features require a high input power to produce acoustic fields that are strong enough to manipulate the specimens, which might damage biological samples.^20,21^ Additionally, depending on their design and arrangement, polymer-based microfluidic devices containing solid features often rely on hydrophilic surface coatings or require being flushed with surfactants, such as isopropanol or ethanol, to reduce the surface tension and, by that, prevent the unintended formation of air bubbles which would otherwise alter anticipated streaming patterns significantly.^19,20,22,23^ Furthermore, most solid-feature-based designs are limited to the generation of in-plane vortices and are, therefore, unable to provide additional visual insights through the reorientation of the specimen.^24^

To overcome these challenges, microstreaming induced by an oscillating microbubble can be utilized. The streaming patterns of the microbubble can be switched from in-plane to out-of-plane streaming *via* altering the excitation frequency, allowing for controlled three-dimensional rotations of cells and organisms,^4,25−28^ as well as microfluidic applications including pumping or mixing.^29−31^ Due to its high compressibility and the corresponding strong oscillations, the microvortices produced by acoustically activated microbubbles are significantly enhanced compared to the ones caused through the vibration of solid structures.^32−34^ However, the major drawback of microbubbles is their limited temporal stability. If acoustically excited, rectified diffusion leads to the bubble’s growth, which alters its resonance frequency and, by that, the strength and shape of the vortices.^35^ This process can be decelerated using commercially available encapsulated bubbles; however, the instability of the thin polymer membrane of encapsulated bubbles complicates their application for long-term investigations.^36^ Finally, as microbubbles are prone to be trapped in cavities due to hydrophilic/hydrophobic interactions, their dimensions show slight variations between multiple experiments, making it difficult to predict their precise response to acoustic excitation and preventing their use for clinical applications.

In this work, we introduce the embedded microbubble, a novel PDMS-based microstructure combining the low-power advantage of bubble-based acoustic streaming with the temporal stability of acoustically excited solid features. The design of the embedded microbubble consists of a rectangular air chamber which is separated from the fluid channel by a thin PDMS wall. We perform in-depth numerical investigations and quantify the influence of geometrical parameters, such as the PDMS wall thickness or the microbubble’s length, on the acoustic streaming inside the fluid channel and utilize these findings to optimize our device design. The manipulation capabilities of our acoustofluidic lab-on-chip are analyzed through experimental characterizations and the revealed insights are discussed in further detail with respect to the numerical results and analytical derivations. Finally, we highlight our device through the controlled and reliable out-of-plane rotation of single HeLa cells, a task beneficial to improve our understanding of biological processes on the cellular and subcellular level, and demonstrate the relevance of our approach for additional microfluidic applications through its use for mixing, a key step required for a wide variety of biomedical as well as chemical research.

## Materials and Methods

### Governing Equations

The force responsible for moving small (relative to the acoustic wavelength) particles in an acoustic field is called acoustic radiation force. For an inviscid fluid, it is given by the negative gradient of the Gor’kov potential *U*(37)
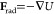
1which can be expressed as
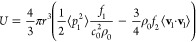
2with the particle radius *r*, the incident acoustic pressure field *p*_1_, the incident acoustic velocity field **v**_**1**_, the fluid speed of sound *c*_0_, the density of the fluid at equilibrium ρ_0_, the monopole *f*_1_ and the dipole *f*_2_ scattering coefficient. ⟨□⟩ denotes time averaging ⟨□⟩ = 1/*T* ∫_*t*_1__^*t*_1_+*T*^ □d*t*, with any point in time *t*_1_ and the period of oscillation *T* = 1/*f*. The Gor’kov potential is derived under the assumption that boundaries are far away from the region of interest, which is not the case here. However, experimental studies revealed that the theory should be valid up to close proximity of the embedded microbubble.^38^

In acoustofluidics, the acoustic energy density is often used as a benchmark for the device’s performance and, therefore, used in our numerical analysis. The average acoustic energy density () is given as^39^
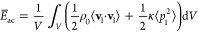
3with compressibility κ and volume *V*.

Another force that needs to be considered in our acoustofluidic chip is acoustic streaming, which affects a particle through a drag force
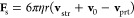
4with the fluid dynamic viscosity η, streaming velocity **v**_str_, background flow **v**_0_, and particle velocity **v**_prt_.

The magnitude of the streaming velocity can be estimated as^40^
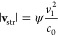
5with the geometry dependant factor ψ = 3/8^41^ for a standing wave parallel to a planar wall.

As can be seen in eqs 1 and 4, the acoustic radiation force and the streaming induced drag force scale with *r* and *r*^3^, respectively. Hence, there exists a critical particle radius at which both forces balance each other. The particle radius can be estimated by^40^
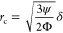
6with the acoustic contrast factor Φ and the viscous boundary layer δ. The acoustic contrast factor Φ can be written as
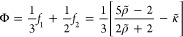
7in which relative compressibility  and density  reflect the ratios between particle (□)_p_ and fluid (□)_f_ (or (□)_0_) properties. In the case of yeast cells dispersed in water, Φ ≈ 0.07.

The viscous boundary layer can be written as
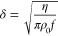
8

Given our excitation frequency of approximately 70 kHz, the boundary layer is δ ≈ 2 μm. When plugging the computed values for δ and Φ into eq 6, we end up with a critical particle radius of *r*_c_ ≈ 6 μm for yeast cells, leading to a streaming dominated behavior. For polystyrene (PS) particles, the acoustic contrast factor increases to Φ ≈ 0.17 leading to a lower critical particle radius of *r*_c_ ≈ 4 μm. Hence, PS particles with 10 μm in diameter are dominated by the acoustic radiation force, while slightly smaller yeast cells, due to their different contrast factor, can be successfully applied to visualize the streaming pattern.

### Numerical Model

We built a two-dimensional (2D) numerical model of the chip and evaluated it in COMSOL Multiphysics (version 5.4). We specifically studied the frequency response of the device. At resonance frequencies, we looked at the wall displacement as well as the Gor’kov potential and streaming velocity. First, a mesh study was conducted to determine the converged mesh parameters (see Figure S1). After the initial frequency-domain study of the Thermoviscous Acoustics and Solid Mechanics interface, a stationary study of the Creeping Flow interface at the resonance frequency was carried out by taking the solutions from the first study into account. With this study, we were able to investigate the streaming velocity. Please refer to the Supporting Information ST1 for a more detailed description of the numerical model.

### Device Fabrication

The poly(dimethylsiloxane) (PDMS) device was fabricated using photolithography (see Figure S2) as well as a Bosch process with an inductively coupled plasma deep reactive ion etching (ICP-DRIE) tool (PlasmaPro 100 Estrelas, Oxford Instruments, United Kingdom). The etching mask consisted of a 7 μm thick AZ nLOF 2070 layer. The effect of prominent reactive ion etching (RIE) lag was reduced through iterative parameter optimizations following an approach introduced by Lai et al.^42,43^ (see Supporting Information ST2 and Figure S3). The etch depth was 47.8 ± 0.7 μm. It is worth noting that other fabrication methods, such as soft lithography *via* SU-8 molds, have been tested. However, due to the unreliable processing of 5 μm narrow trenches with an aspect ratio of 1:10 in a negative photoresist, the DRIE-based approach has been chosen for the final design.

The processed silicon wafer was coated with silane (1*H*,1*H*,2*H*,2*H*-perfluorooctyltriethoxysilane, abcr GmbH, Germany) to ensure successful PDMS casting and prevent damage to the fragile polymer features. Subsequently, designed structures were transferred into PDMS through molding and chemically bonded to a microscope glass slide (AA00000102E01, VWR International, Switzerland) *via* oxygen plasma treatment. During this step, the air located in the chamber gets trapped automatically as the thin PDMS wall separates the chamber from the channel. However, given the permeability of PDMS, the trapped air is not pressurized. Finally, the transducer (KPEG-126, Kingstate, Taiwan) was fixed onto the glass slide using epoxy glue (UHU plus schnellfest, UHU Holding GmbH, Germany). It is worth noting that prefabricated devices demonstrated to be suitable for long-time storage. However, possible changes in acoustic capabilities due to the oxidation of the transducer’s piezoelectric material, a factor which is not limited to our device, have to be considered.

### Experimental Evaluation

Preliminary experiments to find the best excitation frequencies *via* attraction forces were performed with 10.29 ± 1.01 μm fluorescence polystyrene particles (FSEG008, Bangs Laboratories, Germany). When strong resonances were found, yeast cells, i.e., *Saccharomyces cerevisiae*, bought from a local supermarket, were submerged in deionized water and injected into the microfluidic channel to be used as tracer particles for the acoustic streaming. Given the limited visual accessibility of the out-of-plane vortices, experimental evaluation relied on secondary in-plane streaming visualized by the yeast cell circulation near the embedded microbubbles.

The piezoelectric transducer has been excited *via* an arbitrary function generator (AFG3011C, Tektronix). The motion of yeast cells has been captured at 12–17 frames/s using an inverted microscope (IX81, Olympus, Japan). To account for the varying size of yeast cells, multiple frames of the recordings were averaged. Typical in-plane vortices obtained with yeast cells for different excitation voltages are shown in Figure S4b. All data required for individual qualitative evaluations of design or process parameters have been collected in single experimental sessions to ensure comparability and to prevent interdevice discrepancies such as in the distance between the transducer and the PDMS device.

Our device’s mixing capabilities have been evaluated using normalized gray-scale analysis, with a standard deviation threshold of 10% denoting sufficient mixing.^44^ Mixing has been performed using a constant excitation frequency of 69 kHz. Steady volume flows of 0.3 and 0.66 μL min^–1^ for deionized water and black ink (4001, Pelikan, Switzerland) were achieved by syringe pumps (neMESYS, Cetoni, Germany).

To visually inspect the vibration of the 5 μm PDMS wall, it has been recorded with a high-speed camera (CHRONOS 1.4, Kron Technologies, Kanada) at 40 000 frames/s using an inverted microscope (Eclipse Ti, NIKON, Japan) in combination with a 12 V 100 W Halogen bulb in a precentered lamphouse (D-LH/LC, NIKON, Japan) at maximum intensity.

### Biological Model

HeLa S3 cells were cultured in RPMI 1640 (R5886, Sigma Aldrich) with 1× GlutaMAX (35050061, Invitrogen), supplemented with 10% (v/v) fetal calf serum (CVFSVF0001, Eurobio Scientific, France), 100 μg mL^–1^ penicillin/streptomycin (30-002-CI, Corning), and MEM nonessential amino acid solution (100×) (11140050, Gibco). The cells were grown at 37 °C with 5% CO_2_ in a humidified incubator. Prior to their insertion into the chip, cells were detached *via* the addition of 0.05% Trypsin-ethylenediamine tetraacetic acid (EDTA) (1×) (25300096, Gibco).

## Results and Discussion

### Design Optimization and Geometric Analysis

**Figure 1:**
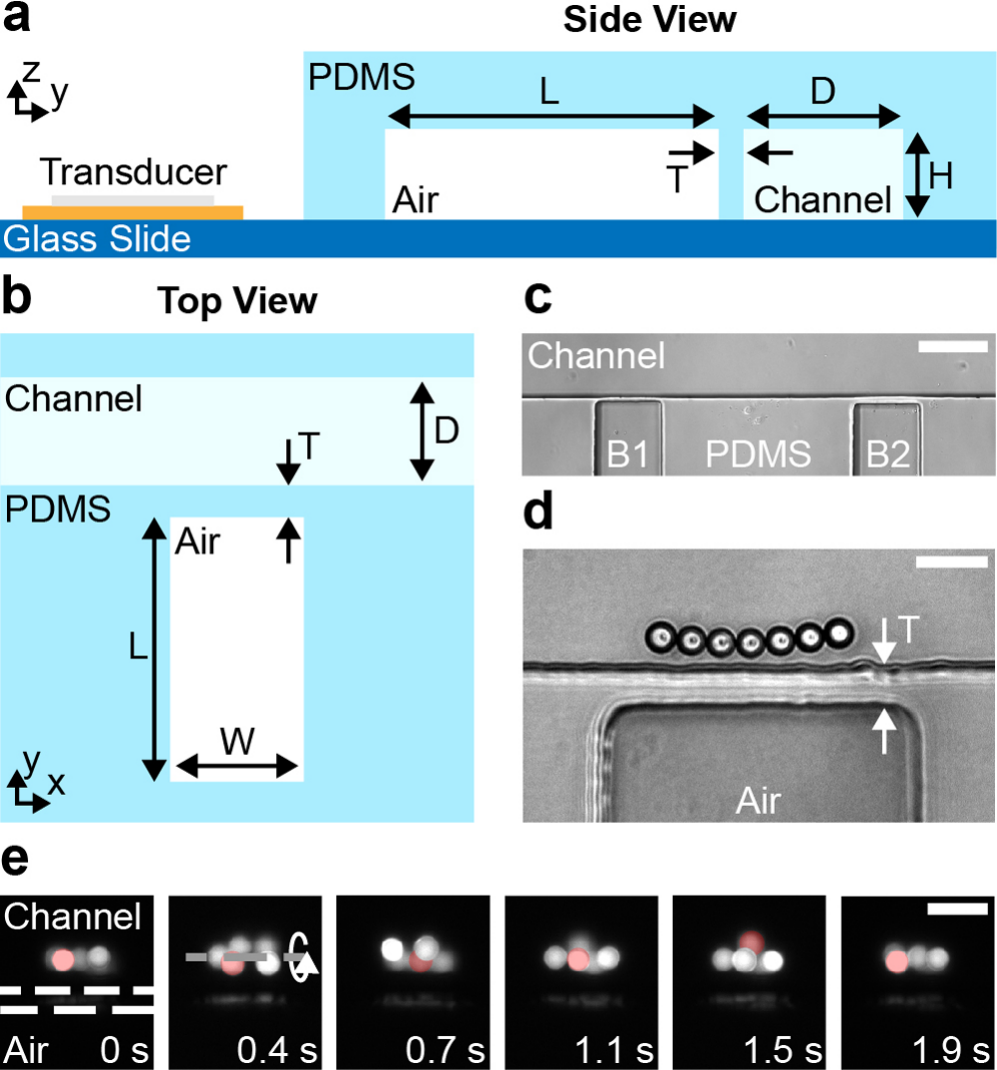
Device layout and microscope pictures of particle attraction and rotation. (a) Side view of a single embedded microbubble. The air trapped in a cavity is separated from the microchannel by a thin wall with thickness *T*. Four designs with different wall thicknesses *T* = 5–20 μm have been fabricated. Additionally, the air chamber length *L* has been varied from *L* = 500 to 1000 μm. The height H of all features is approximately 50 μm. (b) Top view of a single embedded microbubble with the numerically investigated wall thickness *T*, air chamber length *L*, and channel dimension *D*. The width *W* of the embedded microbubble was kept at 100 μm. (c) Two embedded microbubbles B1 and B2 next to each other along the microchannel. B1 and B2 have a wall thickness *T* of 5 and 10 μm, respectively. (d) Seven 10 μm PS particles trapped near an embedded bubble with *T* = 15 μm. The particles are slowly rotating out-of-plane. Particles were only trapped close to an embedded microbubble and not near the channel walls in general. (e) Fluorescent microparticles following an out-of-plane vortex in front of an embedded microbubble. A single particle is highlighted in red as it follows the streaming for two rotations. The white dashed lines indicate the location of the thin PDMS wall and the dashed gray line indicates the center of rotation. Scale bars: *c* = 100 μm, *d* = 25 μm, and *e* = 25 μm.

Accurate investigations and optimizations of design parameters are essential to ensure efficient and reliable manipulation capabilities in lab-on-chip applications. Figure 1 introduces our acoustofluidic device through the side (a) as well as the top (b) view. The main component consists of a poly(dimethylsiloxane) (PDMS) feature that contains a microfluidic channel with dimension *D* = 300 μm as well as embedded microbubbles. The microbubbles have a constant width of *W* = 100 μm but varying lengths *L* and are separated from the nearby liquid channel through thin PDMS walls with different thicknesses *T* = 5–20 μm. The PDMS device is excited *via* a piezoelectric transducer. By driving the transducer at specific frequencies, the thin PDMS wall between the microfluidic channel and the embedded microbubble can be brought to resonate in its eigenmodes, which subsequently allows for the attraction and rotation of small specimens in the nearby liquid.

To investigate the influence of various geometrical design parameters onto the manipulation capabilities of embedded microbubbles, features with different wall thicknesses *T* = 5–20 μm and air chamber lengths *L* = 500–1000 μm have been fabricated and the strengths of the resulting acoustic effects have been evaluated qualitatively. For a device height *H* of 50 μm, a smallest wall thickness of *T* = 5 μm was chosen, which leads to an aspect ratio *H*/*T* = 10. Despite the small thickness of the PDMS wall, no gas diffusion from the air chamber to the liquid channel has been observed that could have led to the formation of free air bubbles.^45^Figure 1c shows two embedded microbubbles aligned along a microchannel with wall thicknesses *T* of 5 μm (B1) and 10 μm (B2). The initial analysis of our device revealed only minor changes in resonance frequencies between the different designs, a factor crucial for the subsequent in-depth experimental, analytical, and numerical investigations.

Figure 1d shows polystyrene (PS) particles trapped near the acoustically excited microstructure. The wall thickness *T* of the embedded microbubble is 15 μm and excitation parameters are 67.1 kHz and 10 *V*_PP_. The 10 μm PS particles remained in their stable positions while slowly rotating out-of-plane. It is worth noting that the particles in the microchannel are only trapped near the embedded microbubbles while not attracted by the regular walls of the microchannel, indicating that the effect is not induced through the general PDMS–liquid interface. In contrast to previous work describing the acoustic streaming near such boundaries,^46,47^ this deviation is likely caused by the different geometries of the channels as well as the applied excitation frequencies and voltages. Figure 1e presents an image sequence of out-of-plane rotating PS particles close to an embedded microbubble with a wall thickness *T* of 15 μm (white dashed lines in the first image). The excitation frequency and voltage are 68 kHz and 10 *V*_PP_, respectively. The demonstrated motion of microparticles is further presented in Supporting Information SV1, which highlights our feature’s capability to generate stable out-of-plane vortices reliably, a crucial task for single-cell analysis as well as the investigation of small model organisms.

**Figure 2:**
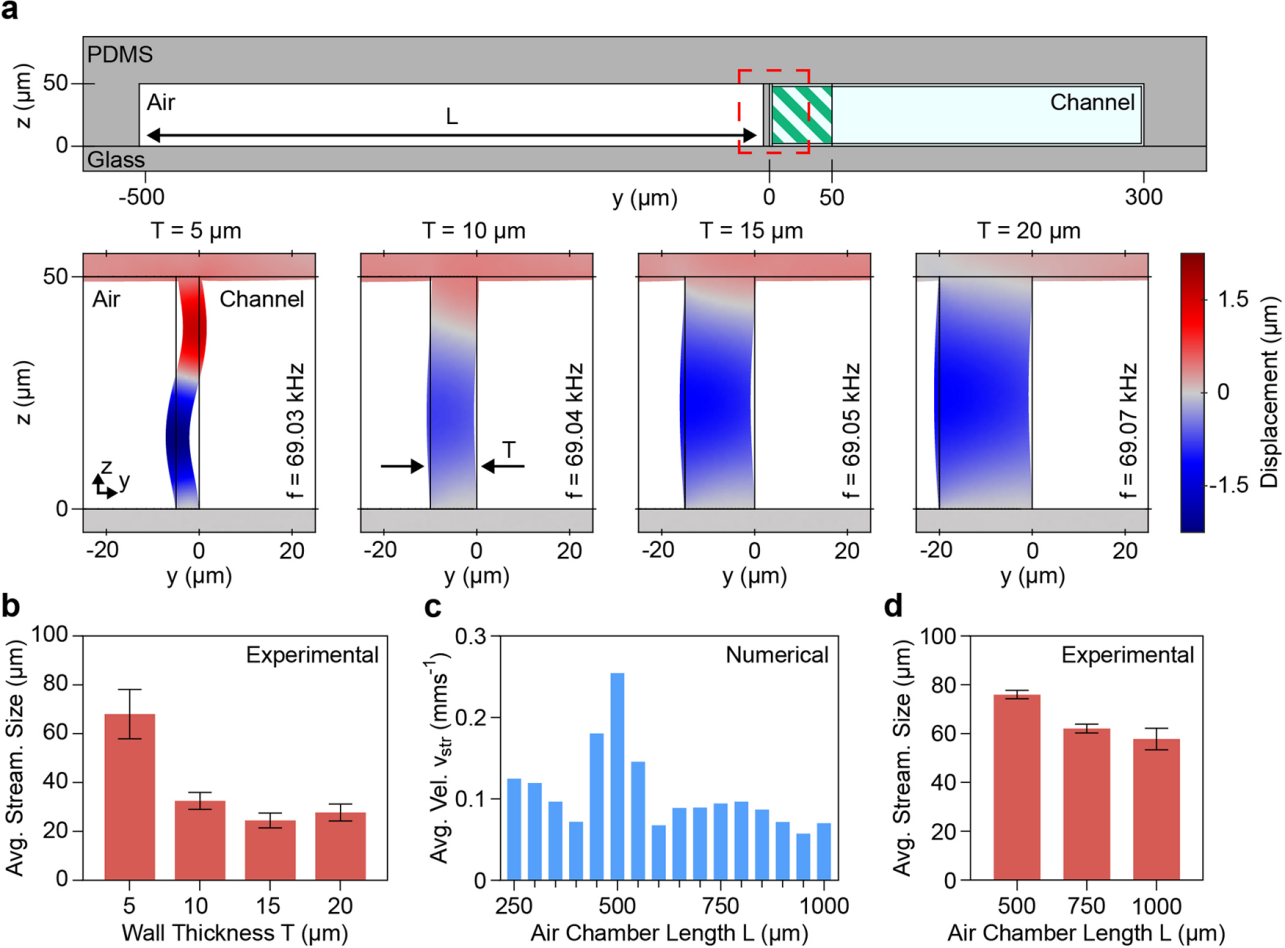
Design optimization with numerical and experimental investigations. (a) Numerical model of the device’s *yz*-plane zoomed in to highlight the embedded microbubble and water channel. The region of interest, containing the thin PDMS wall, is highlighted with a red square. The green hatched area near the PDMS wall inside the fluid channel denotes the area used for the numerical evaluation of the average acoustic energy density *E*_ac_ as well as the average streaming velocity *v*_str_. The wall displacement for varying wall thicknesses *T* = 5–20 μm have been derived through numerical simulations. While thinner walls lead to stronger displacements, similar resonance frequencies of the thicker walls have been observed, indicating that the resonance of the bubble dominates. (b) Experimentally determined in-plane streaming sizes (in *y*-direction) for different wall thicknesses *T* with a constant air chamber length *L* = 500 μm. The largest streaming pattern has been observed for a wall thickness of *T* = 5 μm. (c) Numerical results for the average streaming velocity *v*_str_ at the resonance frequency for air chamber lengths *L* = 250–1000 μm with a constant wall thickness *T* = 5 μm. The maximum average streaming velocity in the area close to the vibrating wall is achieved for an air chamber length of *L* = 500 μm. (d) Experimental results of the in-plane streaming size for different air chamber lengths *L* and a constant wall thickness *T* = 5 μm. The experimental results nicely fit the findings of the numerical investigations, indicating that *L* = 500 μm leads to the largest streaming vortices.

Following our preliminary experimental analysis, we performed numerical investigations of the various designs to optimize the device’s performance. Figure 2a shows the simulated wall displacements for varying wall thicknesses *T*. As our investigations focused on the region near the embedded microbubble, the streaming velocity (eq 5) and the acoustic energy density (eq 3) have been integrated and averaged over the hatched area of 50 × 50 μm. The simulation results suggest that the wall thickness and wall displacement are inversely proportional with thinner walls demonstrating higher displacement amplitudes than thicker walls for the same excitation voltage. The simulations have been performed at each structure’s corresponding mode with the largest displacement, which correlates nicely with the highest acoustic energy density. The modes have been chosen based on preliminary experimental observations that suggested strong acoustic interactions for excitations around a frequency of 69 kHz. Additionally, the resonance frequency of the 5 μm thin PDMS wall has been verified through high-speed imaging (see the Materials and Methods section). Supporting Information SV2 presents the vibration of the wall throughout a frequency sweep from 68 to 70 kHz with the peak of the embedded microbubble’s resonance clearly visible at 68.9 kHz. Unfortunately, due to the significantly reduced displacement for PDMS walls with thicknesses larger than *T* = 5 μm, this evaluation method is unsuitable for detecting the precise modes of the corresponding designs, and, hence, for quantitative purposes, the experimental investigations relied on tracer particles. Additionally, due to vibration being induced along the *z*-direction as well as the high excitation frequency, a detailed visualization of the resonance shape is prevented. Nevertheless, the obtained data is sufficient to provide visual feedback regarding the location of strongest displacement, which can be found at the center of the wall. For further accessibility, Supporting Information SV3 presents the PDMS wall of an embedded microbubble when the excitation is switched from off to on, simplifying the evaluation of the displacement’s spatial distribution. Surprisingly, the numerical simulations revealed only minor variations of the frequencies (69.05 ± 0.02 kHz) with the strongest displacement for the different wall thicknesses *T*, which indicate that the amplitude of the wall’s displacement is not solely dependant on the wall thickness but might additionally be influenced by the geometry of the air, water, and PDMS components. This assumption was further verified by an extended frequency sweep for all wall thicknesses. Figures S5–S7 display the typical S-shaped resonances of different geometries. It is important to note that, despite being in resonance, the amplitudes of their vibrations decrease for increasing excitation frequency. Since the decrease in displacement is mainly independent of the wall’s geometry, the investigated vibrations are likely affected by the resonances of the air chambers of the embedded microbubbles. Using the following equation, the Minnaert frequency,^48^ i.e., the natural frequency of a spherical microbubble, can be derived as^49^
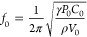
9with the polytropic coefficient γ, the bubble’s gas pressure and volume *P*_0_ and *V*_0_, respectively, its capacitance *C*_0_, which contains the frequency’s dependency and the bubble shape, and the mass density of the surrounding liquid ρ.

Our microbubble with *L* = 500 μm, *W* = 100 μm, and *H* = 50 μm can be approximated by a prolate spheroid with the same volume using an eccentricity ϵ derived as
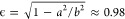
10where *a* ≈ 49 μm stands for the semiminor axes along *x* and *z* and *b* = *L*/2 = 250 μm is the semimajor axis along the *y*-direction.

Therefore, the ratio of the electrostatic capacitance of our prolate spheroid *C*_p_ and a spherical microbubble with the same volume *C*_0_ can derived as^50^
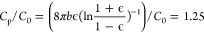
11where *C*_0_ = 4π*R*_0_ with the radius of the spherical microbubble *R*_0_ = *b*(1 – ϵ^2^)^1/3^ ≈ 85 μm.

Finally, the Minnaert–Strasberg frequency for our prolate spheroid can be calculated as
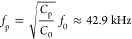
12for a natural frequency *f*_0_ ≈ 38 kHz of a spherical bubble with equal volume in water at standard pressure. Please note that while the initial simplification of our geometry to a prolate spheroid might lead to a shift in resonance frequency, the bubble’s frequency is generally expected to show only a slight dependency on the precise aspect ratio.

The determined Minnaert–Strasberg frequency can be further adjusted to include the influence of the surrounding PDMS (see Supporting Information ST3) as well as including a frequency shift induced by a rigid wall such as the glass slide enclosing our design (see Supporting Information ST4).^51^ However, both adjustments introduce only minor corrections to the previously calculated natural frequency of a prolate microbubble and, hence, are insufficient to describe the numerically as well as experimentally observed maximum resonances of the embedded microbubbles. The obtained results, therefore, highlight the complex relationship between the various geometrical parameters while shedding light on a possible dependency or interaction between the resonances of different structural components, which influences the overall manipulation capabilities of our device.

Subsequently, we performed experimental characterizations of the strong dependence between wall thickness and wall displacement highlighted by the numerical simulations. As a measure for comparison between different geometries, yeast cells have been introduced into the microchannel as tracer particles. The sizes of the resulting in-plane streaming vortices were taken as a measure for comparison (see Figure S4). A channel dimension *D* = 300 μm has been chosen to avoid interference between the local acoustic streaming at the embedded microbubble and the opposing wall. As can be seen in Figure 2b, significantly larger streaming patterns have been achieved for embedded microbubbles with 5 μm walls than for designs with thicker features. It is important to highlight that all embedded microbubbles were located on the same device and arranged next to each other along the microchannel, as shown in Figure 1c, to prevent possible inaccuracies in the results due to variations in the setup. Additionally, all features have been evaluated around an excitation frequency of 66 kHz, i.e., at their corresponding resonance frequency, and a voltage of 20 *V*_PP_ with only minor distinctions between resonance peaks being possible through visual observations. Excitation of the device at significantly higher or lower frequencies did lead to none or only minor acoustic streaming, indicating that the wall has a limited activity at other frequencies as expected by our numerical analysis (see Figures S5–S7). While no clear differences in the streaming patterns have been observed for the wall thicknesses of *T* = 10–20 μm, higher input power might be required to allow for a clear distinction of the influence of this geometrical parameter.

Following the quantification of the wall thickness’ effect on the acoustic capabilities of our device, we proceeded with the investigation of the influence of the air chamber length *L*. In numerical simulations, the air chamber length *L* has been increased from 250 to 1000 μm with steps of 50 μm. Figure 2c indicates that the strongest average streaming velocity can be expected for *L* = 500 μm. At this air chamber length, the surrounding PDMS seems to compress the embedded air in a way that supports the vibrational mode of the resonating thin wall. To provide further insights into the complex relationship between the resonance of the embedded microbubble, the wall thickness *T*, and the air chamber length *L*, the major modes detected in a frequency range of 40–125 kHz have been numerically analyzed for varying air chamber lengths, i.e., for *L* = 250, 500, 750, and 1000 μm. Figure S8a shows a frequency sweep of an embedded microbubble with *T* = 5 μm and *L* = 500 μm with its corresponding resonances. The detailed investigations for the different air chamber lengths *L* at these resonances (see Figure S8b) revealed that a modification in air chamber length *L*, in general, induced a minor frequency shift in the range of 1 kHz or less. However, the varying resonance peaks did not follow a specific order, such as small air chamber length *L* leading to lower resonance frequencies or *vice versa*. Additionally, the numerical simulations showed that the strongest resonances of the embedded microbubble with regard to the wall displacement and the average acoustic energy density *E*_ac_ were obtained for excitation frequencies around 41 and 69 kHz despite inducing different resonance shapes of the PDMS wall along the *z*-direction. Finally, it has been observed that, irrespective of the air chamber length *L*, all excitation frequencies above the 69 kHz resonance lead to significantly lower responses of our design and a reduction of its acoustic manipulation capabilities.

Therefore, it is likely that, while the resonance of the embedded microbubble is a complex construct that depends on various parameters simultaneously, its manipulation capabilities are maximized for designs that ensure similar resonance frequencies for the air chamber, as controllable through an appropriate air chamber length *L*, and the PDMS wall, for which the thickness *T* is expected to be of major relevance. Given this conclusion and the high number of air bubble-based resonances, it is advisable to focus on the vibration of the wall as key parameter during the initial characterization of novel devices. However, other factors, such as large-scale deformations of the PDMS device due to overall changes in the stability of the features cannot be fully excluded. Subsequently, the influence of the air chamber length *L* has been quantified experimentally (Figure 2d). All microstructures have been excited using the same parameters, i.e., frequency and voltage, and the sizes of the generated in-plane vortices have been measured. While the experimentally obtained results expose a similar trend as predicted by the numerical simulations, the superiority regarding the streaming size of *L* = 500 μm is less pronounced in experiments; therefore, further investigations might be necessary to allow for the appropriate interpretation of these results. A non-negligible reason for the variation observed between the numerically determined results and the experimental quantifications might also be based on differences in the evaluated quantity. While the numerical simulations allow for a direct investigation of the streaming velocity of the produced out-of-plane streaming near the embedded microbubble, due to limited accessibility to this streaming during experimental evaluations, the latter has to rely on the generated in-plane vortices. Therefore, the comparison of the results obtained through different techniques, i.e., numerical and experimental evaluations, might be restricted by the complex and possibly nonlinear relationship between the out-of-plane and the in-plane streaming vortices.

### Investigation of Acoustofluidic Capabilities

**Figure 3:**
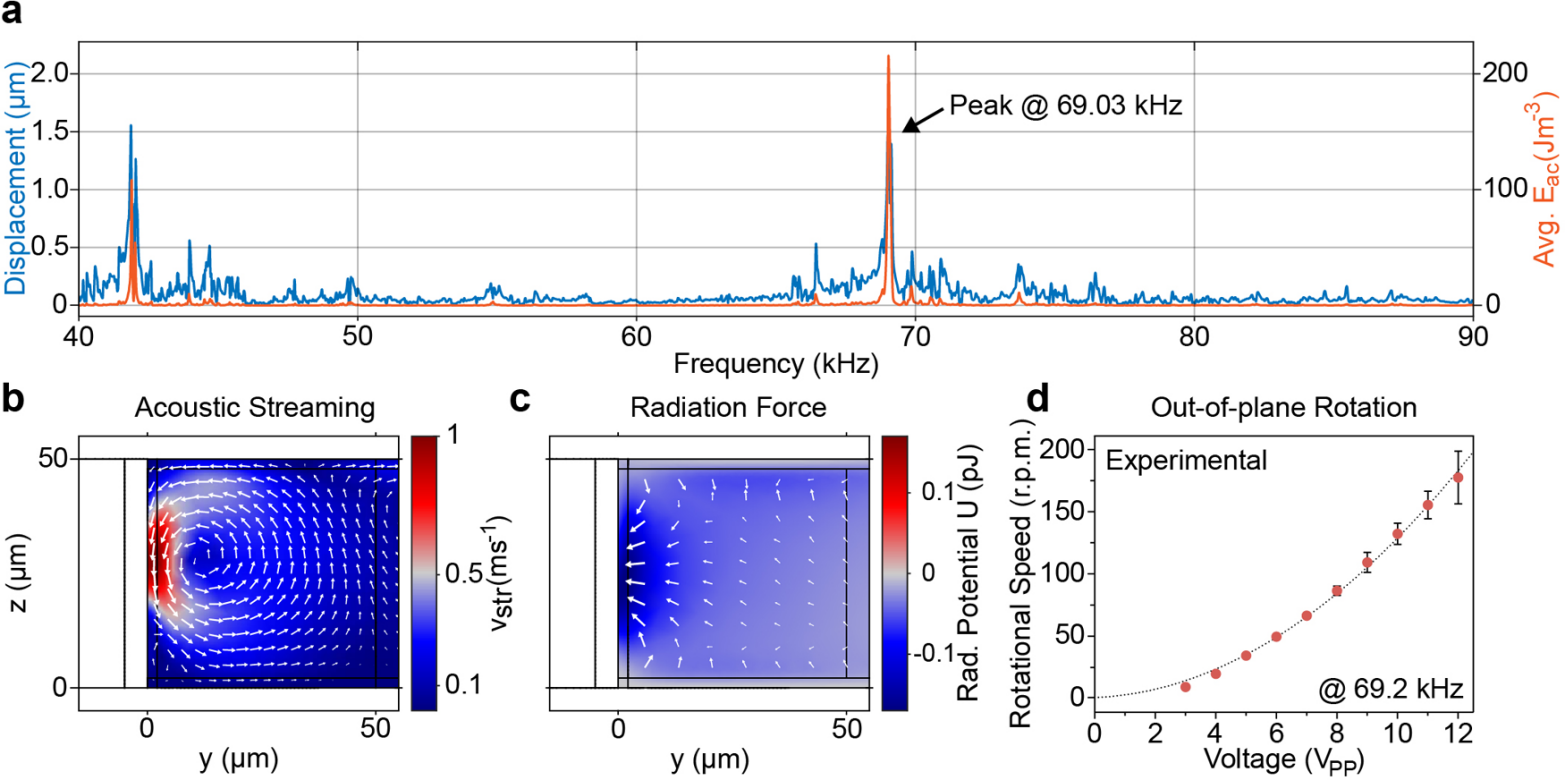
Detailed investigation of a device with wall thickness *T* = 5 μm and embedded microbubble length *L* = 500 μm. (a) Simulated frequency sweep from 40 to 90 kHz with steps of 0.1 kHz. The displacement of the thin PDMS wall and the average acoustic energy density *E*_ac_ show prominent resonances at 41.89 and 69.03 kHz. (b) Simulated streaming velocity field near the thin PDMS wall. The velocity of the acoustic streaming is the strongest close to the wall and drives the fluid in a counterclockwise fashion. (c) Simulated Gor’kov potential as well as the acoustic radiation force (white arrows). The minimum of the Gor’kov potential can be found at half the height of the thin wall. Therefore, the acoustic radiation force is pointing toward this position, which, in experiments, leads to a particle attraction from the channel toward the embedded microbubble. Both (b) and (c) were derived at an excitation frequency of 69.03 kHz which corresponds to the strongest resonance. (d) Experimentally determined relationship between the excitation voltage and the out-of-plane rotational speed of a specimen (rotation around *x*-axis). While the excitation frequency is kept constant at 69.2 kHz, an increase of the input power leads to stronger streaming velocities (eq 5) and subsequently to faster rotations of the specimen. The dashed line denotes a fitted quadratic curve with a coefficient of determination *R*^2^ = 0.98.

Based on the results presented in the device optimization, we opted for a design with wall thickness *T* = 5 μm and air chamber length *L* = 500 μm and performed more specific numerical analyses to gain further insights regarding the underlying mechanisms and the acoustic phenomena. Figure 3a shows the average wall displacement and the average acoustic energy density for frequencies from 40 to 90 kHz with 100 Hz steps. The maximal energy density correlates with the highest displacement of the wall and reveals two strong resonances (41.89 and 69.03 kHz). While Figures 2a and 3b,c demonstrate the behavior of our system at an excitation frequency of 69.03 kHz, Figure S9 presents the resonance of the embedded microbubble through the simulated vibration of the PDMS wall at an excitation frequency of 41.89 kHz as well as the resulting out-of-plane acoustic streaming and Gor’kov potential with the corresponding radiation force. The analysis at 41.89 kHz reveals that, due to the different mode shape of the PDMS wall, the acoustic streaming generated in the nearby liquid consists of two smaller vortices compared to the streaming near 69 kHz. While the lower resonance frequency also nicely fits the derived natural resonance, i.e., the Minnaert–Strasberg frequency, of the prolate spheroid as calculated in eq 12, numerical as well as experimental investigations showed that frequencies around 69 kHz lead to much stronger particle attraction and streaming velocities than frequencies around 40 kHz. The small deviation between the experimentally observed and the simulated resonance frequency can be attributed to idealized material parameters and the limited significance of the displacement boundary condition, e.g., through lower amplitudes of the piezoelectric element at this frequency range. However, the aim of the numerical investigations is not to attain exact amplitudes but to show trends and get insight into the physical phenomena of the device. Furthermore, it is important to highlight that the manipulation capabilities of the embedded microbubble, as demonstrated through the previous numerical, analytical, and experimental investigations, are likely the result of a complex interplay between various parameters including the volume of the air chamber, which is linked to the air chamber length, as well as the PDMS wall thickness and the wall’s resonance mode.

At the resonance frequency of 69.03 kHz, a strong fluid vortex is generated close to the thin wall that drives the fluid in a counterclockwise fashion, ranging up to 50 μm into the channel (see Figure 3b), and could be utilized for controlled particle rotation. However, the particle needs to be positioned close to the thin wall to be influenced by the streaming vortex, which can be achieved by the acoustic radiation force. For particle attraction toward the thin wall, the Gor’kov potential minimum needs to be close to the embedded microbubble (see eq 1). As can be seen in Figure 3c, at the resonance frequency of 69.03 kHz, a Gor’kov potential minimum is generated at half of the height of the thin wall; thus, particles are attracted to the displacement node of the thin PDMS wall. It is important to highlight that the generated node is not based on a standing wave formation inside the channel and, thereby, does not rely on the geometrical dimensions of the PDMS as a whole.^52^

Figure 3d shows the experimentally evaluated relationship between the out-of-plane rotational speed of a specimen and the applied excitation voltage. The embedded microbubble has been excited at a constant frequency of 69.2 kHz, which is in great accordance with the numerically detected maximal wall displacements around that frequency. The rotational speed follows the input voltage in a quadratic dependency (*R*^2^ = 0.98), demonstrating our approach’s high controllability of the specimen orientation which is crucial for biomedical applications such as single-cell analysis. It is important to note that the increasing error at high voltages might be induced through uncertainties based on the frame rate limitations of the experimental setup. However, additional sources, including possible cell membrane instabilities or the motion of intracellular features during fast rotational manipulations, cannot be excluded.

### Single-Cell Manipulation and Microfluidic Mixing

We expand the investigation of our device by demonstrating its application for two diverse applications, i.e., single-cell manipulation as well as microfluidic mixing. HeLa cells are a prominent model organism for biomedical as well as genetic research^53,54^ and their controlled manipulation exposes great potential to allow for further insights on a single-cell level.^5^

**Figure 4:**
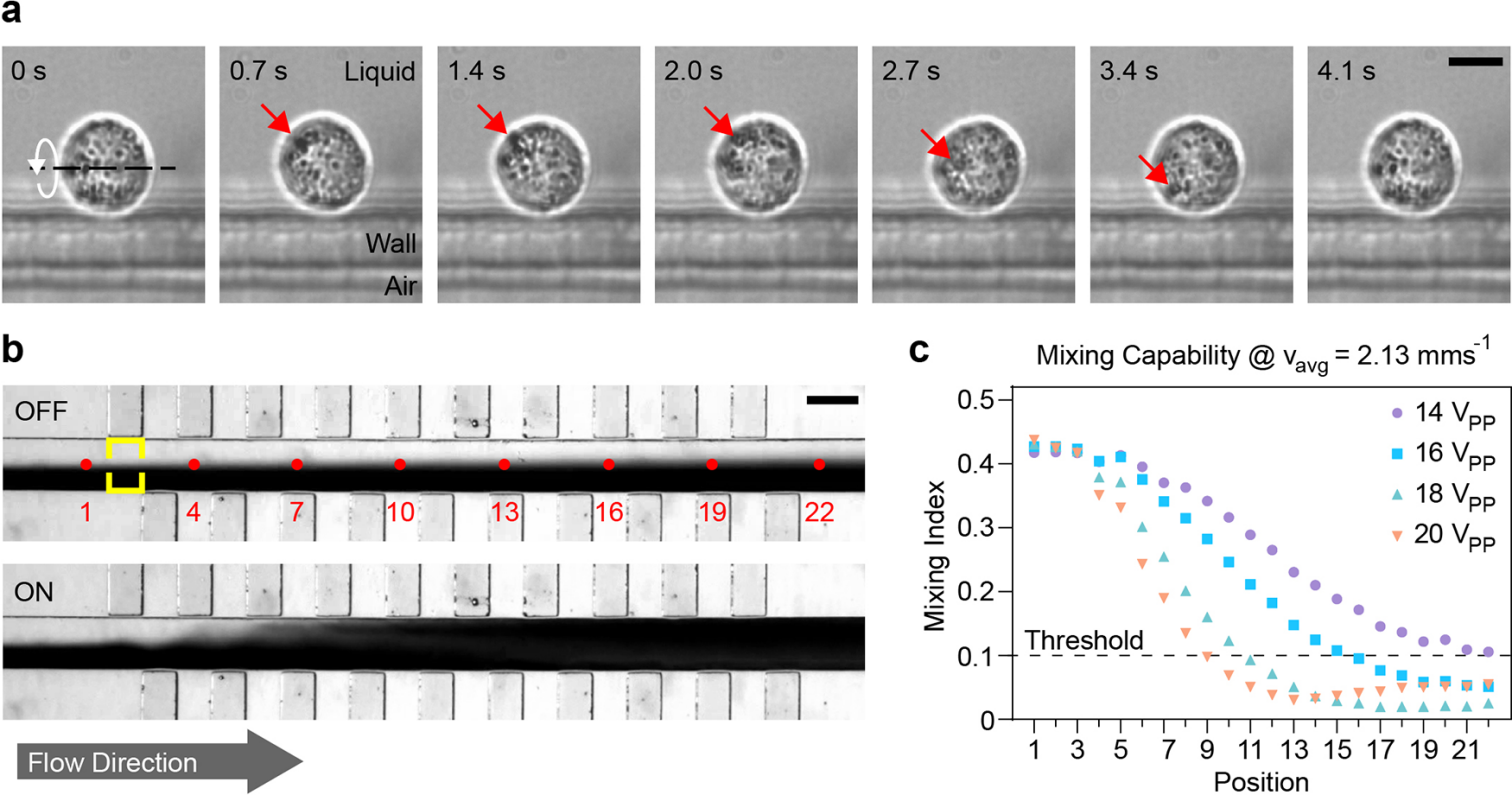
Microfluidic applications for embedded microbubbles. (a) Controlled out-of-plane rotation of a single HeLa cell. The microstructure is excited at a constant frequency and voltage of 69.2 kHz and 5 *V*_PP_, respectively. The white arrow denotes the direction of the specimen’s rotation, while the red arrow highlights a spot on the specimen’s surface. (b) Microfluidic mixing of two liquids in a 150 μm wide microchannel based on acoustically activated embedded microbubbles. The top image shows the laminar flow of the liquid where mixing is limited to diffusion. The red dots show the positions along the microchannel used for the evaluation of the mixing efficiency. The yellow rectangle indicates the area used for the calculation of the mixing index at position 2. The bottom image demonstrates the mixing procedure for an excitation voltage of 20 *V*_PP_ and a flow velocity of *v*_avg_ = 2.13 mm s^–1^. (c) Experimental evaluation of the mixing index for different input voltages. The mixing index corresponds to the standard deviation of the normalized gray-scale image at the corresponding positions of the microchannel. The horizontal line at 10% denotes sufficient mixing. Scale bars: (a) = 10 μm; (b) = 150 μm.

The image sequence in Figure 4a presents the slow and stable out-of-plane rotation of a single HeLa cell near an embedded microbubble with a wall thickness *T* = 5 μm while the piezoelectric transducer is excited with a constant frequency of 69.2 kHz and a voltage of 5 *V*_PP_. The HeLa cell’s motion is further presented in Supporting Information SV4. Please note that, in contrast to many solid microstructures used for acoustic manipulation,^24^ the demonstrated rotational motion has been achieved at a low excitation voltage of 6 *V*_PP_, thereby preventing possible damage to the sample due to high-power pressure fields. The importance of input power has recently been highlighted by Wang et al.,^21^ where the application of 30 *V*_PP_ input power, in combination with their PDMS-based sharp-edge design, led to the lysis of approximately 60% of exposed Jurkat and HeLa cells, and cell lysis was increased to nearly 100% for an excitation voltage of 50 *V*_PP_. Hence, single-digit excitations are preferable for the manipulation of biological specimens. The successful manipulation of the HeLa cell using our device is based on the combination of the acoustic radiation force and acoustic streaming. As demonstrated through the numerical simulations, the Gor’kov potential (see Figure 3c) leads to a force which pushes biological specimens toward the embedded microbubble. Once trapped, the single cell is rotated *via* the viscosity-related acoustic streaming, which allows for the visual investigation of the specimen’s intracellular components as well as individual features of the cell membrane. Assuming the HeLa cell to be spherical, the resulting viscous torque acting on the specimen during a rotation at an excitation voltage of 5 *V*_PP_ can be approximated *via* the Stokes’ drag as^55^
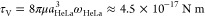
13where μ = 1 mPa is the dynamic viscosity of water at room temperature, *a*_HeLa_ = 8 μm is the radius of the HeLa cell, and ω_HeLa_ = 3.48 rad s^–1^ is its rotational speed.

To further demonstrate the manipulation capabilities of our device, Figure S10 presents the out-of-plane rotations of a larger and shape-anisotropic HeLa cell for varying excitation voltages, i.e., for 4 and 10 *V*_PP_. It is worth highlighting that the sample maintained a stable rotational motion despite its bigger size and irrespective of the orientation of its major axis. Please note that the wall thickness appears larger in the image sequences due to the plane of focus being set on the specimen. Additionally, given the structural limitation induced by the channel height *H* = 50 μm, the manipulation of even larger objects, such as zebrafish embryos or larvae,^2,56^ is not possible with the current design and would likely require further fabrication improvements.

Due to the flow limitations at the low Reynolds regime, reliable mixing is essential for a variety of lab-on-chip applications in chemical as well as biomedical analysis.^57−59^ Our acoustic-based mixer consists of a series of alternating embedded microbubbles with wall thickness *T* = 5 μm arranged along a single microchannel. The channel dimension *D* has been set to 150 μm based on numerical investigations to ensure strong and local streaming near the microstructures. Two liquids, i.e., deionized water and black ink, are introduced through separate channels, while their volume flow is controlled *via* a high-precision microfluidic pumping system to achieve an average flow velocity *v*_avg_. The inlet channels meet at an angle of 90° to minimize diffusion-based mixing while avoiding possible acoustic streaming near sharp-edge features.^14,20,60^

Figure 4b shows the PDMS device with the two laminar flows prior to acoustic excitation, i.e., the piezoelectric transducer is off, as well as during mixing of two fluids using an input voltage of 20 *V*_PP_ (see Supporting Information SV5). The average flow velocity inside the microchannel is *v*_avg_ = 2.13 mm s^–1^, which, despite requiring significantly lower input power and not relying on surface treatments, is comparable to results presented for solid structure-based acoustic mixers.^44,61,62^ Additionally, as our design allows to prevent geometrical constrictions, unintended trapping of microbubbles inside the mixing channel can be avoided. While bubble-based acoustic mixers allow for increased handling of fluid volumes, our approach circumvents their limitations in temporal stability and reusability.^29,63,64^ The graph in Figure 4c demonstrates the experimentally determined relationship between the applied input power and the acoustic manipulation capabilities of our device. To allow for the evaluation of device performance, we calculated mixing indices at different positions of the microchannel, starting with position 1 in front of the mixer (see red labels in Figure 4b). For each position, a mixing index has been derived as the standard deviation of the normalized gray-scale image (area highlighted with yellow lines). A threshold of 10% has been defined as sufficient mixing based on previous literature.^44^ The graph demonstrates that for our design with 20 alternating embedded microbubbles and for average flow velocities *v*_avg_ = 2.13 mm s^–1^, successful mixing can be achieved for excitation voltages as low as 16 *V*_PP_ (blue squares), while for 20 *V*_PP_ (orange triangle), the threshold is already reached after eight features.

To allow for direct comparison between various designs and techniques applied in microfluidic mixing, the average mixing time can be derived as
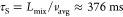
14where *L*_mix_ = 800 μm is the mixing distance required to achieve sufficient mixing for an excitation voltage of 20 *V*_PP_. However, it is important to note that the device’s efficiency might be further improved through design optimizations, such as by arranging the embedded bubbles in an opposite instead of an alternating manner. Additionally, as active streaming has only been observed directly in front of the embedded microbubbles, the distance between the features could be reduced without leading to unintended and possibly unfavorable interactions between the generated vortices. Furthermore, through the application of an external amplifier, the strength of the acoustic streaming could be further increased, which would allow to reduce the derived mixing distance. However, given that the goal of lab-on-chip technology lies in the miniaturization of individual tools, the addition of an amplifier suitable for the necessary frequency range can significantly increase the complexity of the overall system and, by that, might represent an additional obstacle in transferring the demonstrated method to clinical and industrial applications. Nevertheless, it is important to highlight that, following the simplified exponential relation observed between the excitation voltage and the rotational speed as shown in Figure 3d, already a small increase in the input power can have a non-negligible influence on the resulting mixing performance of the microfluidic device.

## Conclusions and Outlook

In this work, we introduced a novel acoustofluidic device and demonstrated its use for particle and single-cell manipulation as well as microfluidic applications. The embedded microbubble combines the advantages of solid features and bubble-based devices, such as their temporal stability, out-of-plane manipulation capability, and low power consumption, while minimizing their drawbacks. Furthermore, in contrast to various acoustic PDMS-based devices relying on solid structures, no surface treatments are required to prevent the unintentional trapping of air bubbles. We numerically investigated different geometrical design parameters, derived their complex relationship to the acoustically generated streaming pattern, and compared the results to the analytically obtained natural frequencies of microbubbles. Then, following noticeable fabrication improvements, we successfully confirmed our observations through experimental characterizations. While optimal device performance with regard to its out-of-plane rotation capabilities is expected to rely on the close relation between nearby resonance frequencies for individual components, the numerical investigation revealed the maximum efficiency for a microbubble length of 500 μm, wall width of 5 μm, and fluid channel width of 150 μm.

We explored the applicability of our device for biomedical research through the controlled out-of-plane rotation of single HeLa cells and quantified the near quadratic dependency between the applied voltage and the specimen’s rotational speed, which allows for the high controllability necessary to achieve slow yet stable motions crucial for future biological investigations, e.g., through fluorescence imaging. Finally, we illustrated our lab-on-chip subsecond mixing performance and discussed potential design improvements for increased efficiency. Nevertheless, it is worth highlighting that the current achievements are comparable to previous publications while relying on significantly lower input power and maintaining the microbubble’s temporal stability.

Future work may include mixing-based gradient generation important for various chemical applications, the improvement of microfluidic mixing performance through the combination of embedded microbubbles and sharp-edge structures,^65^ and the extension of our approach for manipulating multicellular organisms, such as *Caenorhabditis elegans*.

## Supporting Information Available

Mesh study of numerical simulation (Figure S1), illustration of the fabrication process (Figure S2), etch lag improvement (Figure S3), experimental evaluation approach (Figure S4), higher-order vibration for *T* = 10 μm (Figure S5), higher-order vibration for *T* = 15 μm (Figure S6), higher-order vibration for *T* = 20 μm (Figure S7), influence of air chamber length *L* (Figure S8), resonance near 41 kHz for *T* = 15 μm and *L* = 500 μm (Figure S9), rotation of a shape-anisotropic HeLa cell for varying voltages (Figure S10), parameters of a numerical model (Text ST1), etch lag reduction approach (Text ST2), Minnaert–Strasberg frequency in PDMS (Text ST3), Minnaert–Strasberg correction for rigid boundary (Text ST4) (PDF)
Polystyrene particle manipulation (Video SV1) (AVI)
Vibration of the PDMS wall during frequency sweep (Video SV2) (AVI)
Vibration of the PDMS wall with excitation from OFF to ON (Video SV3) (AVI)
Stable HeLa cell rotation (Video SV4) (AVI)
Acoustic mixing capabilities (Video SV5) (AVI)

## Supplementary Material

ac1c01209_si_001.pdf

ac1c01209_si_002.avi

ac1c01209_si_003.avi

ac1c01209_si_004.avi

ac1c01209_si_005.avi

ac1c01209_si_006.avi
