# Supplementary material for: Embedded Microbubbles for Acoustic Manipulation of Single Cells and Microfluidic Applications
Source: Anal Chem. 2021 Jul 6;93(28):9760–70. doi: 10.1021/acs.analchem.1c01209 (PMC8295982; doi:10.1021/acs.analchem.1c01209)
Supplement: Supplementary file 1 — ac1c01209_si_001.pdf [file ac1c01209_si_001.pdf]

Supporting Information

Embedded Microbubbles for Acoustic Manipulation of Single Cells and Microfluidic Applications

Nino F. Läubli,[1,2,†,*] Michael S. Gerlt,[3,†] Alexander Wüthrich,[1] Renard T. M. Lewis,[4] Naveen Shamsudhin,[1] Ulrike Kutay,[4] Daniel Ahmed,[1,5] Jürg Dual,[3] and Bradley J. Nelson[1]

[1] **Multi-Scale Robotics Lab, Institute of Robotics and Intelligent Systems, Department of Mechanical and Process Engineering, ETH Zurich, Tannenstrasse 3, 8092 Zurich, Switzerland**
[2] **Molecular Neuroscience Group, Department of Chemical Engineering and Biotechnology, University of Cambridge, Philippa Fawcett Drive, Cambridge CB3 0AS, United Kingdom**
[3] **Mechanics and Experimental Dynamics, Institute of Mechanical Systems, Department of Mechanical and Process Engineering, ETH Zurich, Tannenstrasse 3, 8092 Zurich, Switzerland**
[4] **Institute of Biochemistry, Department of Biology, ETH Zurich, Otto-Stern-Weg 3, 8093 Zurich, Switzerland**
[5] **Acoustic Robotics for Life Sciences and Healthcare, Institute of Robotics and Intelligent Systems, Department of Mechanical and Process Engineering, ETH Zurich, Säumerstrasse 4, 8803 Rüschlikon, Switzerland**
† These authors contributed equally to this work.
*** E-mail: laeublin@ethz.ch**

This PDF file includes:

Figure S-1 to S-10
Text ST-1 to ST-4
Legends for Videos SV-1 to SV-5
SI References

Other supporting information for this manuscript include the following:

Video SV-1 to SV-5

Contents

1 Supporting Figures **S3**
- A Figure S-1 . . . S3
- B Figure S-2 . . . S4
- C Figure S-3 . . . S5
- D Figure S-4 . . . S6
- E Figure S-5 . . . S7
- F Figure S-6 . . . S8
- G Figure S-7 . . . S9
- H Figure S-8 . . . S10
- I Figure S-9 . . . S11
- J Figure S-10 . . . S12

2 Supporting Texts **S13**
- A Text ST-1 . . . S13
- B Text ST-2 . . . S13
- C Text ST-3 . . . S13
- D Text ST-4 . . . S14

3 Supporting Videos **S15**
- A Video SV-1 . . . S15
- B Video SV-2 . . . S15
- C Video SV-3 . . . S15
- D Video SV-4 . . . S15
- E Video SV-5 . . . S15

1. Supporting Figures

A. Figure S-1.

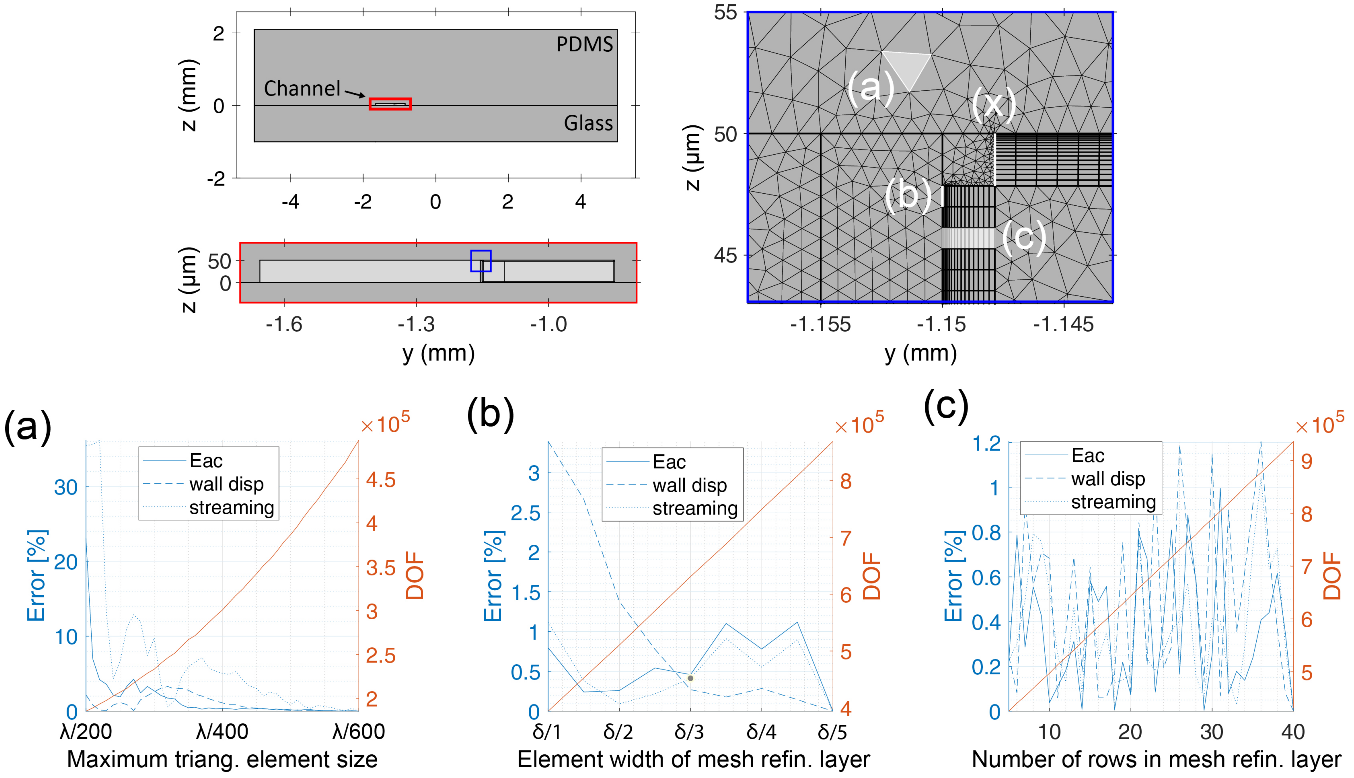

Fig. S-1. Mesh study of the numerical simulation.
We introduced three mesh parameters reflecting different aspects of the mesh. On the top left, we show the numerical model and two magnifications that show the channels and the upper part of the thin wall in between the two channels with mesh, respectively. (a) Maximal triangular element size throughout the whole model given as size per wavelength (λ/n). (b) Width of the elements in the mesh refinement layer as multiples of the viscous boundary layer thickness (δ/n). (c) The number of rows within the mesh refinement layer. The width of the mesh refinement layer (x) was found to be of negligible importance and thus set to the viscous boundary layer thickness $x = \delta$. With E_{ac} as the average energy density in the water channel, wall disp as the average wall displacement of the thin PDMS wall, streaming the average streaming velocity, and DOF the number of degrees of freedom. We chose the following mesh parameters for all simulations in this work, corresponding to an error below $1\,\%$ with respect to the finest mesh: maximum triang. element size = $\lambda/500$ = 5 µm (air), 11 µm (water), 162 µm (glass); element width of mesh refinement layer = $\delta/2.5$ = 0.86 µm; number of rows in the mesh refinement layer =15.

B. Figure S-2.

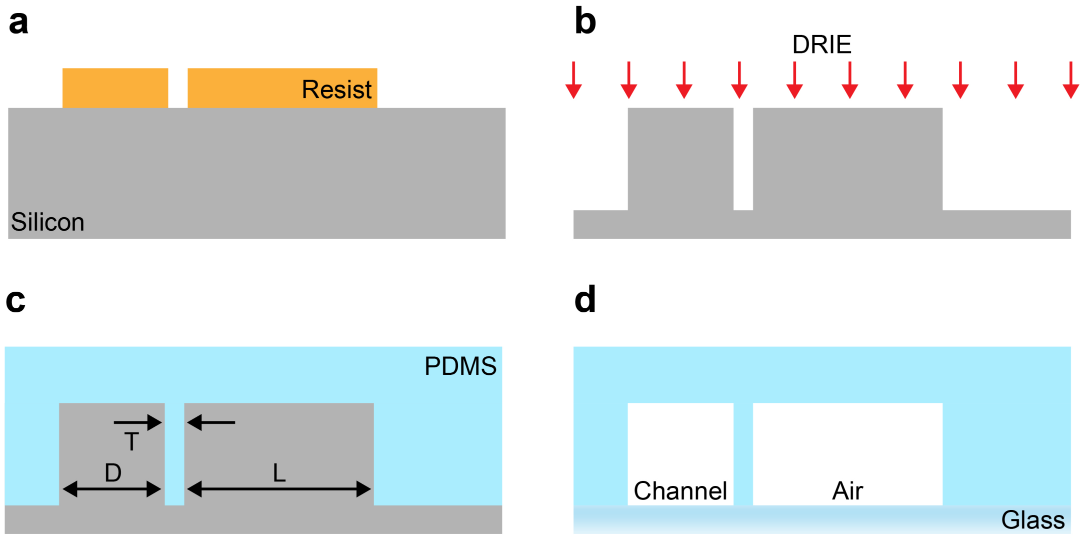

Fig. S-2. Fabrication process for embedded microbubbles.
(a) The design of the embedded microbubble is transferred onto a silicon wafer using photolithography. (b) An optimized DRIE etching process is applied to etch the design into the silicon wafer and produce a mould containing narrow trenches at the locations of the required thin PDMS walls. (c) The wafer is vapour-coated with silane to ensure successful device replication in PDMS casting. The PDMS structures as peeled off the silicon mould and chemically bonded to glass slides using oxygen plasma.

C. Figure S-3.

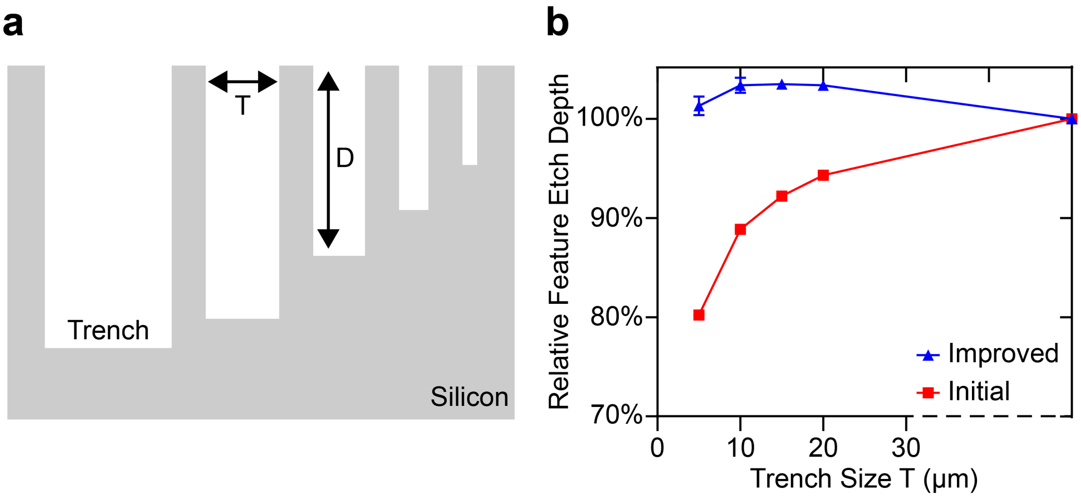

Fig. S-3. Aspect ratio dependent etching (ARDE) lag improvement.
(a) ARDE lag as typically observed for silicon processing. Wide microstructures expose a higher etching rate which subsequently leads to deeper trenches than for narrow channels. (b) The red squares denote the relative etch depths, *i.e.*, the features etch depth in relation to depths achieved in non-constricted areas, for a standard Bosch process. According to this graph, if open spaces of a wafer are etched to a depth of 50 µm, the depth of 5 µm thin trenches will only reach approximately 40 µm, resulting in insufficient bonding for embedded microbubbles with thin walls. The blue triangles present the resulting relative etch depths for a process with significantly reduced ARDE lag. The feature depths have been evaluated using white light interferometry.

D. Figure S-4.

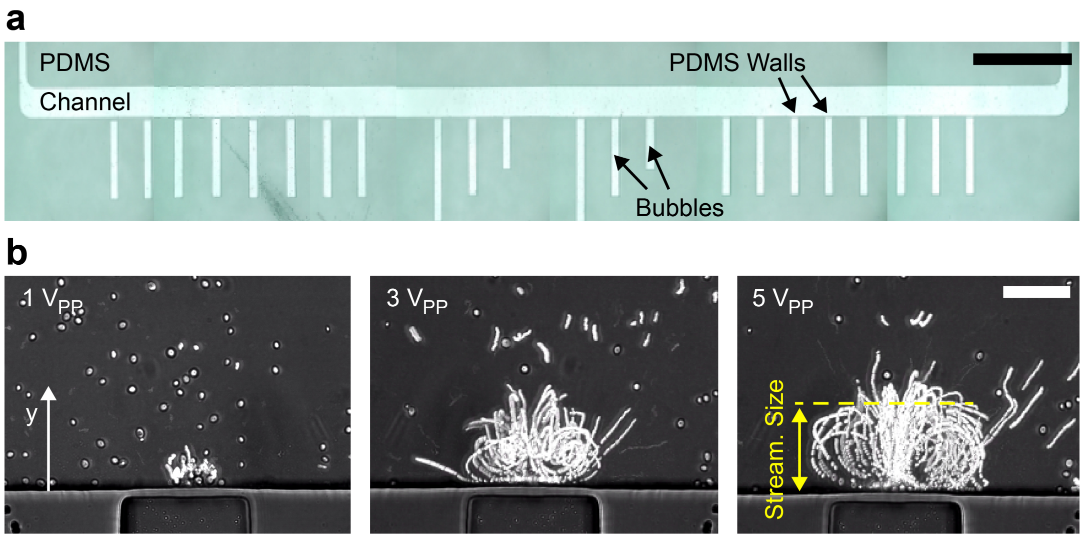

Fig. S-4. Device for experimental characterisation and in-plane streaming visualised by yeast cells.
(a) Stitched optical microscopy image of a PDMS microchannel with different embedded microbubbles used to quantify the effect of various wall thicknesses T and air chamber lengths L. (b) Optical microscopy images resulting from averaging multiple frames of an embedded microbubble with wall thickness $T = 5\,\mu m$ and air chamber length $L = 750\,\mu m$ excited at a frequency of 66.2 kHz and input voltages from 1 V_{PP} (left) to 5 V_{PP} (right). The generated in-plane vortices of the acoustic streaming are visualised using yeast cells. To allow for direct comparison between different inputs as well as varying geometrical properties of the quantified embedded microbubbles, the corresponding in-plane streaming sizes along the y-direction (as shown on the right side) have been determined. Scale bars: (a) = 1 mm, (b) = 50 µm.

E. Figure S-5.

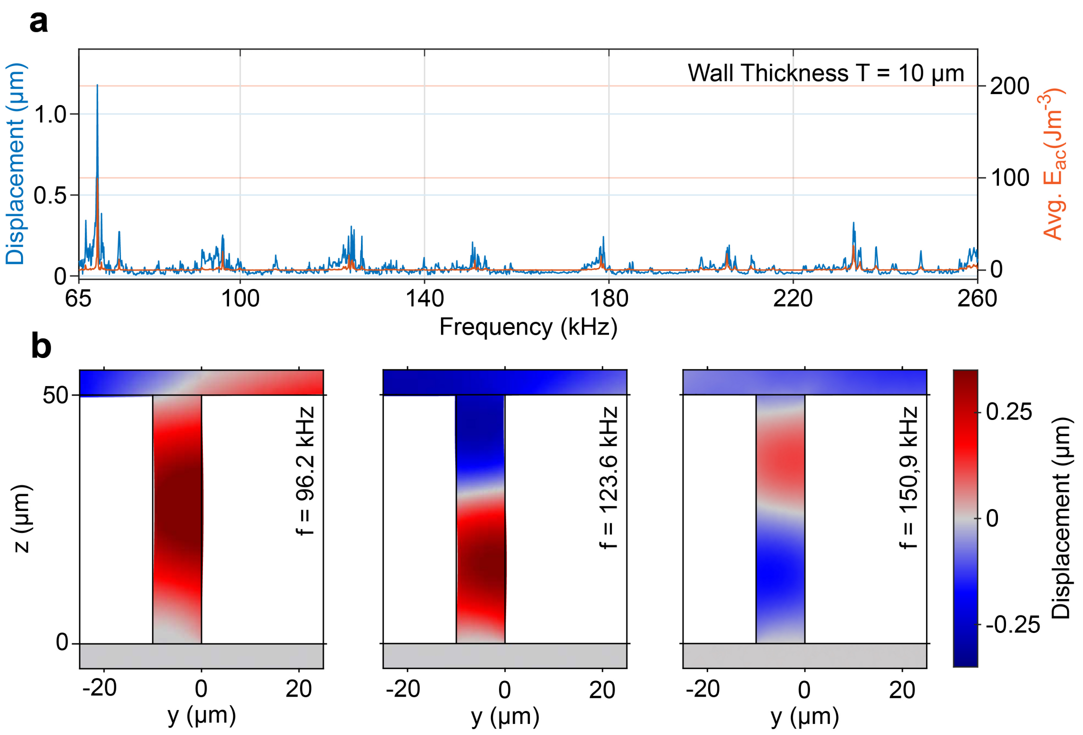

Fig. S-5. Extended frequency study of a device with wall thickness $\mathrm{T} = 10\,\mu\mathrm{m}$ and embedded microbubble length $\mathrm{L} = 500\,\mu\mathrm{m}$. (a) Frequency sweep from 65 to 260 kHz with steps of 0.1 kHz. The displacement of the thin PDMS wall and the average acoustic energy density E_{ac} show resonances at frequencies above 69.04 kHz depicted in Figure 2. (b) Displacement of the wall at selected resonance frequencies. To achieve the same mode shape of the wall as for a wall thickness of T = 5 µm (see Figure 2), one has to excite the wall with T = 10 µm at 123.6 kHz. Slightly lower and higher frequencies lead to a different mode shape.

F. Figure S-6.

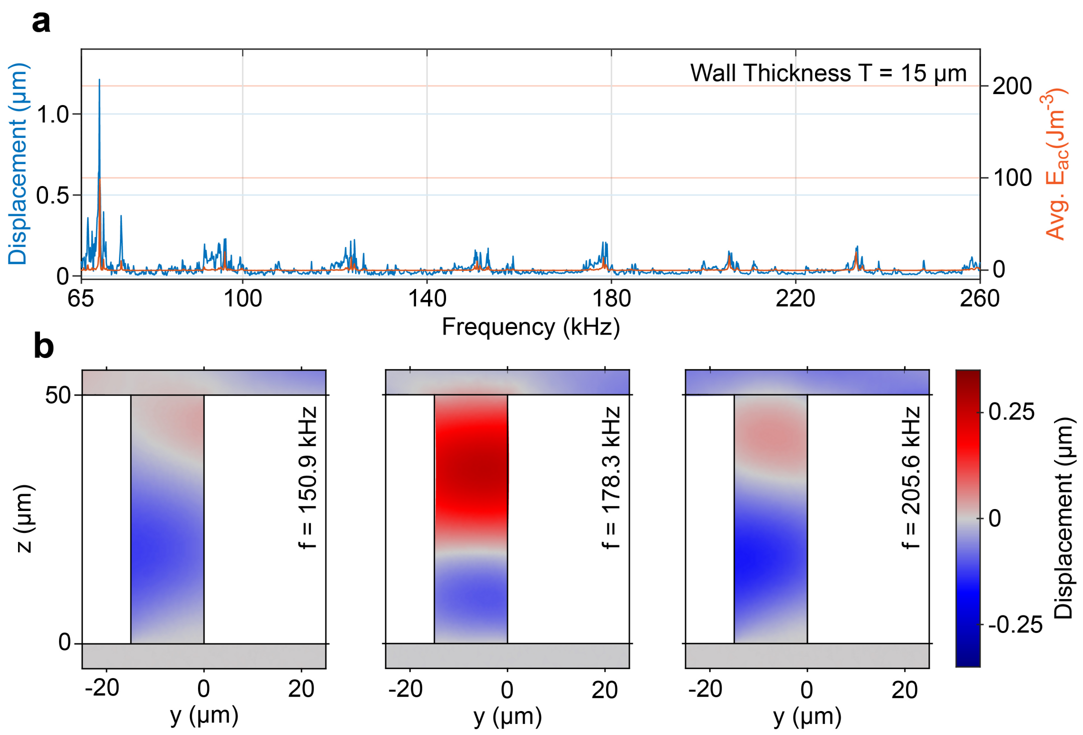

Fig. S-6. Extended frequency study of a device with wall thickness $\mathrm{T} = 15$ µm and embedded microbubble length $\mathrm{L} = 500$ µm. (a) Frequency sweep from 65 to 260 kHz with steps of 0.1 kHz. The displacement of the thin PDMS wall and the average acoustic energy density E_{ac} show resonances at frequencies above 69.05 kHz depicted in Figure 2. (b) Displacement of the wall at selected resonance frequencies. To achieve the same mode shape of the wall as for a wall thickness of T = 5 µm (see Figure 2), one has to excite the wall with T = 15 µm at 178.3 kHz. Slightly lower and higher frequencies lead to a different mode shape.

G. Figure S-7.

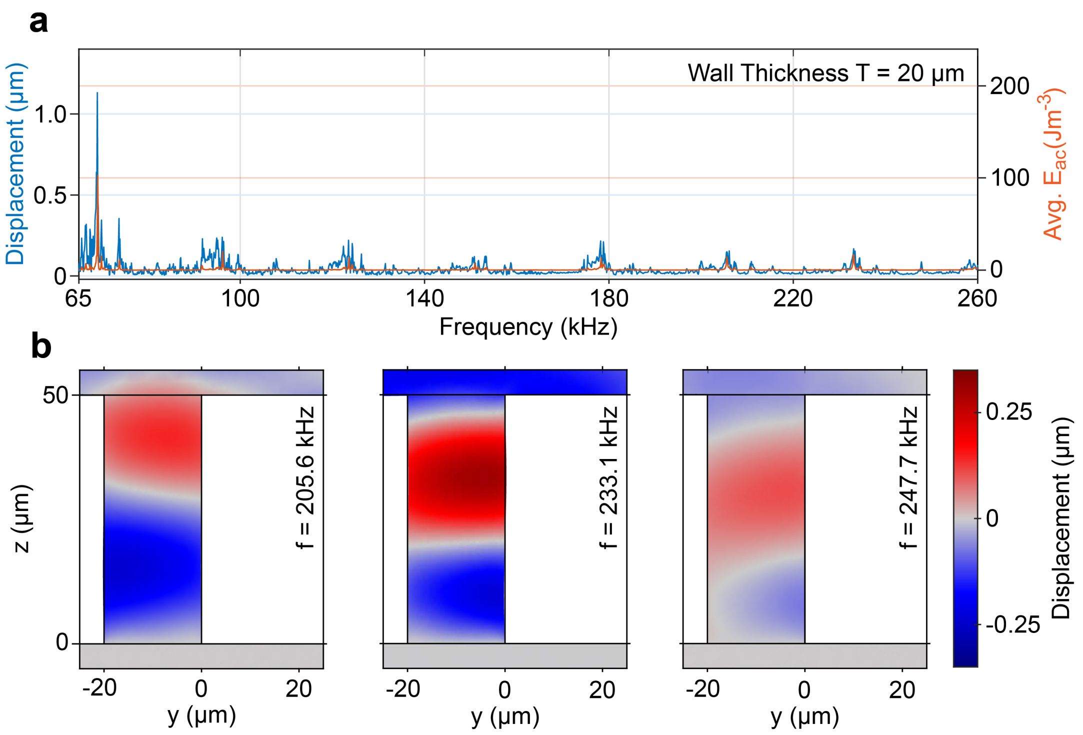

Fig. S-7. Extended frequency study of a device with wall thickness $\mathbf{T} = 20$ µm and embedded microbubble length $\mathbf{L} = 500$ µm. (a) Frequency sweep from 65 to 260 kHz with steps of 0.1 kHz. The displacement of the thin PDMS wall and the average acoustic energy density E_{ac} show resonances at frequencies above 69.07 kHz depicted in Figure 2. (b) Displacement of the wall at selected resonance frequencies. To achieve the same mode shape of the wall as for a wall thickness of T = 5 µm (see Figure 2), one has to excite the wall with T = 20 µm at 233.1 kHz. Slightly lower and higher frequencies lead to a different mode shape.

H. Figure S-8.

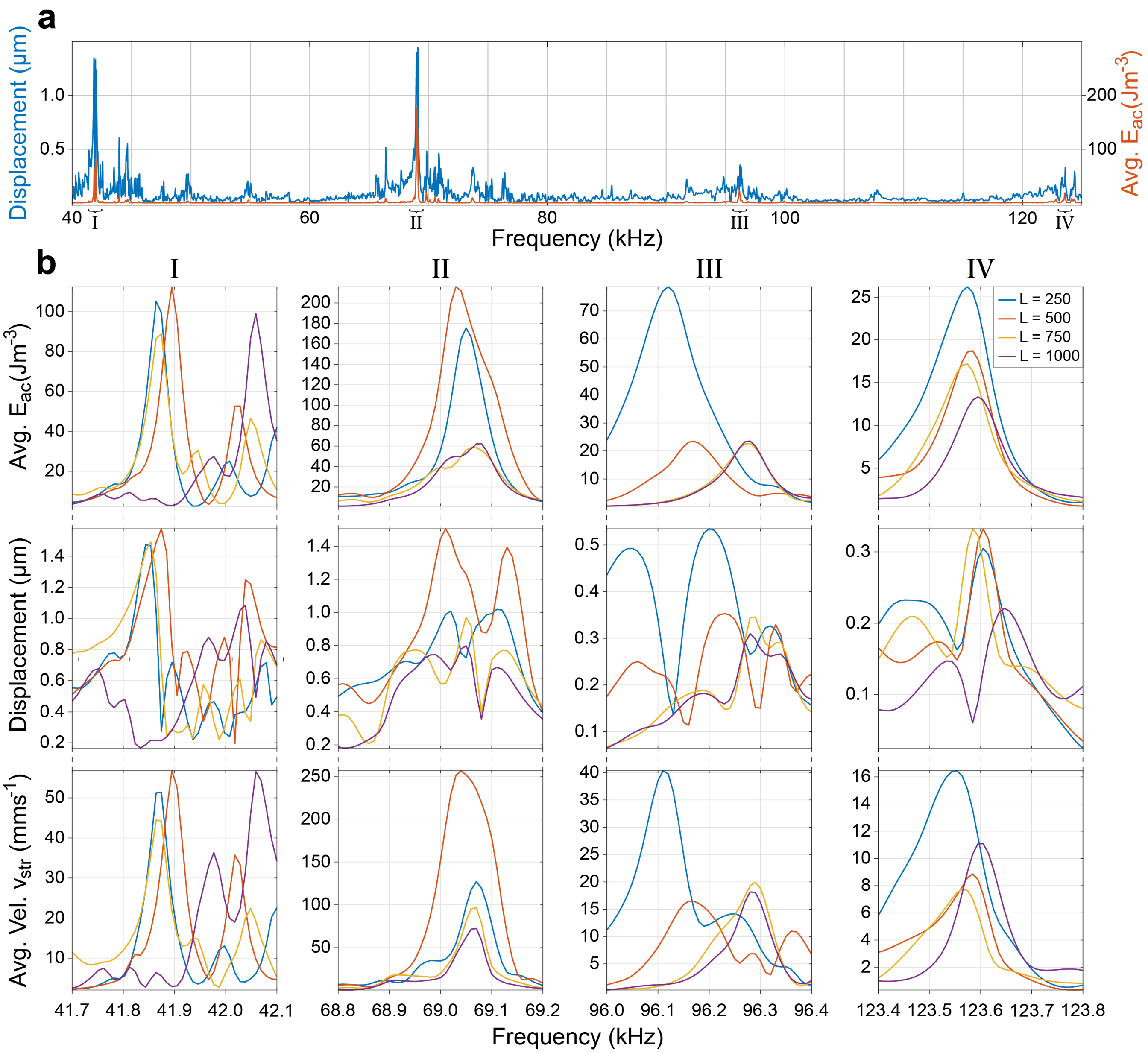

Fig. S-8. Influence of air chamber length $\mathbf{L}$ on resonance.
(a) Frequency sweep of an embedded microbubble with $T = 5\,\mu m$ and air chamber length $L = 500\,\mu m$. The resonances of the embedded microbubble are labelled with Roman numbers. (b) Numerical analysis of the different modes of the embedded microbubble regarding average acoustic energy density E_{ac}, the displacement of the PDMS wall, and the average streaming velocity v_{str} for varying air chamber lengths, *i.e.*, $L = 250 - 1000\,\mu m$.

I. Figure S-9.

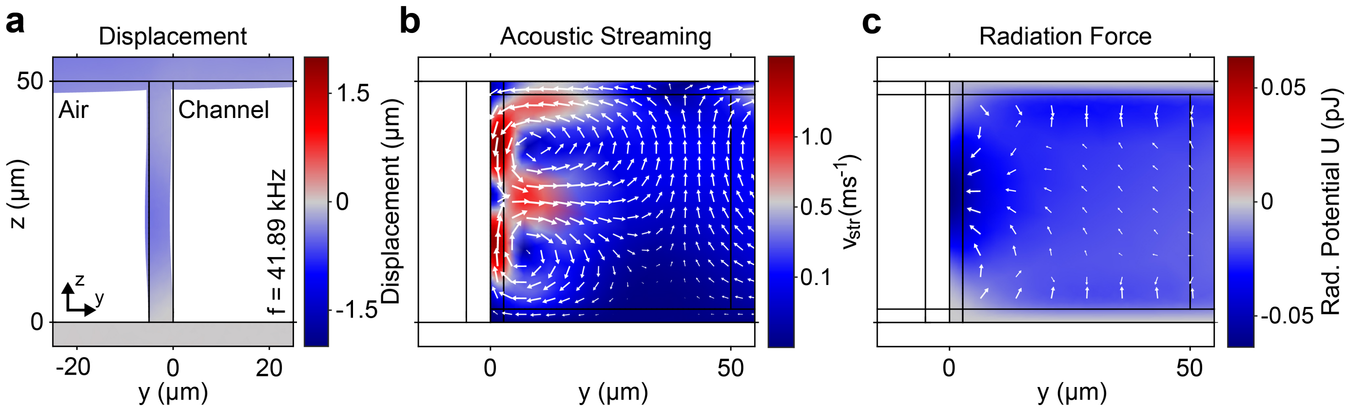

Fig. S-9. Numerical simulation of the resonance at 41.89 kHz of an embedded microbubble with $T = 5\,\mu m$ and $L = 500\,\mu m$. (a) The shape of the resonance mode visualised through the displacement of the PDMS wall. (b) The simulated acoustic streaming near the PDMS wall. In contrast to the embedded microbubble's resonance, the streaming consist of two smaller out-of-plane vortices. (c) The simulated Gor'kov potential near the embedded microbubble and resulting acoustic radiation force.

J. Figure S-10.

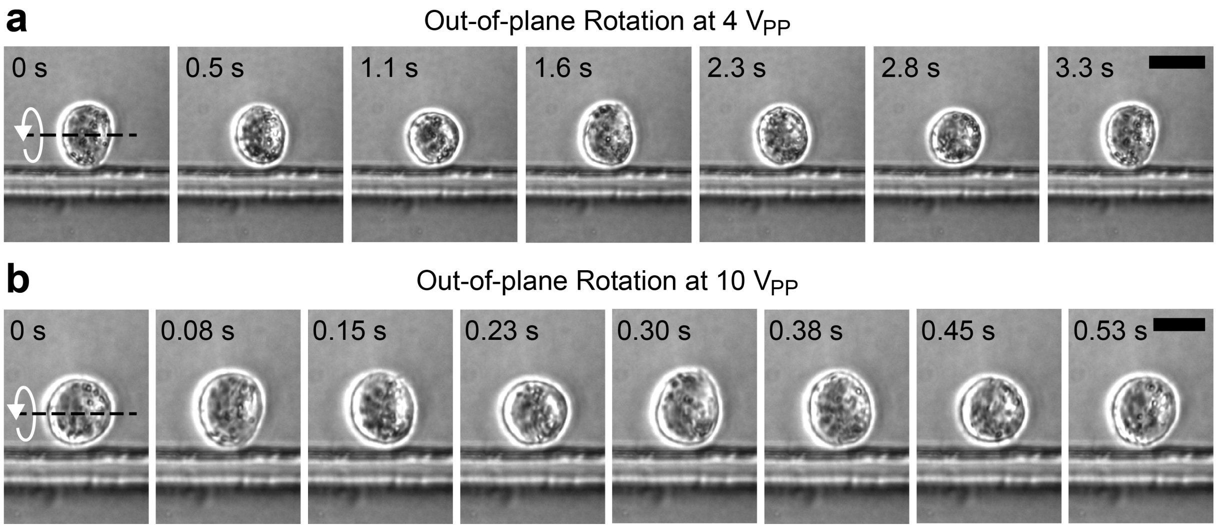

Fig. S-10. Out-of-plane rotation of a HeLa cell with shape anisotropy.
(a) An anisotropically shaped HeLa cell performing a stable out-of-plane rotation at an excitation voltage of 4 V_{PP}. (b) The same HeLa cell performing a stable out-of-plane rotation at an excitation voltage of 10 V_{PP}. The rotation of this HeLa cell has been used to derive the relation between applied excitation voltage and rotational speed as presented in Figure 3 d). Scale bars: (a) = 20 µm, (b) = 15 µm.

2. Supporting Texts

A. Text ST-1.

Supporting Information ST-1: Numerical model

We used the Solid Mechanics interface to model the solid components of the model (glass, PDMS). To account for damping, we chose to model the glass as a linear elastic material with complex Lamé parameters. The PDMS was modelled as a linear elastic material with Poisson's ratio and complex Young's modulus to account for damping in our system. Please refer to table S-1 for all material parameters.

Table S-1. Table of the material properties and damping factors.

Parameter	Symbol and value	Unit
**Glass Pyrex** (1)		
Density	$\rho^{\text{glass}} = 2240$	$[\text{kg m}^{-3}]$
Quality factor	$Q^{\text{glass}} = 2420$	$[\text{kg m}^{-3}]$
Lamé parameters	$\lambda = 23.1 \times 10^9(1 + i/Q^{\text{glass}})$	$[\text{N m}^{-2}]$
	$\mu = 24.1 \times 10^9(1 + i/Q^{\text{glass}})$	$[\text{N m}^{-2}]$
**PDMS Sylgard 184** (2)		
Density	$\rho^{\text{PDMS}} = 970$	$[\text{kg m}^{-3}]$
Poisson ratio	$\nu = 0.49$	[–]
Quality factor[1]	$Q^{\text{PDMS}} = 1000$	[–]
Complex Young's modulus	$E^{\text{PDMS}} = 0.75(1 + i/Q^{\text{PDMS}})$	[MPa]

[1]: this value has been utilised to distinguish between individual resonance peaks. It does not represent the actual Q-factor of the material

The mechanical vibrations were implemented in the model by means of a prescribed displacement boundary condition within the solid mechanics interface. The displacement was applied over the whole length of the bottom of the glass plate and its amplitude was set to 10 nm.

The Thermoviscous Acoustics interface was applied to both channels within the PDMS. The materials inside both channels were modelled with the standard material parameters provided by the software.

The Thermoviscous Acoustic-Structure Boundary interface was implemented to couple the Solid Mechanics interface and the Thermoviscous Acoustics interface at the Water-PDMS and the Air-PDMS interfaces.

To incorporate acoustic streaming in our simulations, we followed the procedure described in (3, 4). We assigned the Creeping Flow interface to the water domain. Volume Forces were set on the whole domain. Finally, a Pressure Point Constraint was set to an arbitrary point of the domain boundary ($p_0 = 0[Pa]$).

B. Text ST-2.

Supporting Information ST-2: ARDE lag reduction

Aspect ratio dependent etching (ARDE) lag has been reduced through iterative parameter optimisations. Figure S-3 a) shows ARDE lag as typically observed in deep reactive ion etching, where wider trenches are etched deeper than narrow structures. This observation is further represented by the red squares in Figure S-3 b) denoting the etch depth for trenches with varying widths T in relation to the depth D achieved in unconfined areas of the processed wafer. Following an approach introduced by Lai *et al.*, (5) we adjusted the durations of the two process steps, *i.e.*, of t_D for the deposition or passivation and t_E for the etching, to allow for equal etch rates between trenches with varying widths and, subsequently, the reduction of ARDE lag (see blue triangles in Figure S-3 b)). It is important to highlight that the derived durations are closely related to the aspect ratio dependency of the individual steps, with the polymer deposition and silicon etching being mostly chemically induced while the initial polymer etching at the bottom of the trench relies on physical processes.

C. Text ST-3.

Supporting Information ST-3: Minnaert-Strasberg frequency in PDMS

To account for the PDMS environment, the derivation of the free spherical microbubble presented in the manuscript can be adjusted as follows: (6)

$$f_{\text{PDMS},0} = \frac{1}{2\pi}\sqrt{\frac{\gamma P_0 C_0}{\rho_{\text{PDMS}} V_0}}, \qquad [1]$$

where γ is the polytropic coefficient, P_0 is the bubble's gas pressure, V_0 is the bubble's volume, C_0 is the capacitance, and ρ_{PDMS} is the density of the surrounding PDMS.

By assuming equal conditions and volumes for the spherical bubbles in water and PDMS, the natural frequency of a spherical bubble in PDMS can be calculated as

$$f_{\text{PDMS},0} = f_0\sqrt{\frac{\rho_{\text{Water}}}{\rho_{\text{PDMS}}}} \approx 38.6\,\text{kHz}, \qquad [2]$$

with the previously derived natural frequency of the free bubble in water $f_0 \approx 38\,\mathrm{kHz}$, the density of water $\rho_{\mathrm{Water}} = 1000\,\mathrm{kg\,m^{-3}}$, and the density of PDMS $\rho_{\mathrm{PDMS}} = 970\,\mathrm{kg\,m^{-3}}$.

As the change in the surrounding material properties does not influence the electrostatic capacitance of our microbubbles, the natural resonance frequency of a prolate spheroid in PDMS with a volume equivalent to an embedded microbubble with air chamber length $L = 500\,\mathrm{\mu m}$ can be derived as (7)

$$f_{\mathrm{PDMS,P}} = \sqrt{\frac{C_P}{C_0}}\, f_{\mathrm{PDMS,0}} \approx 43.2\,\mathrm{kHz}, \qquad [3]$$

with a capacitance ratio $C_P/C_0 = 1.25$ as presented in the manuscript.

While the change in surrounding media only let to a minor adjustment of the of the bubbles resonance frequency, it is important to highlight the possibly limited significance of this derivation, as the initial formulations used for the calculation of the bubble's natural frequency are intended for liquid environments and, hence, the results should be evaluated with the necessary caution.

D. Text ST-4.

Supporting Information ST-4: Frequency shift due to a nearby solid wall

As the influence of shape-anisotropy as well as the introduction of PDMS environment have been detected as insufficient to describe the major resonance observed in numerical and experimental characterisations of embedded microbubbles, the analytical derivation is extended through the correction of the resonance frequency for nearby rigid walls, such as the glass slide enclosing our design. The presence of the rigid boundary, *i.e.*, the glass slide, is simulated by the addition of a second spherical microbubble with equal radius R_0. Its centre is located at a distance $2\,d$ from the first one, where d is equal to the bubble radius such that their surfaces are in contact.

As analytically derived by Strasberg in 1953 and numerically confirmed by Spratt in 2017, the capacity for each of these spherical bubbles can be calculated as (8, 9)

$$C_{\mathrm{PDMS,W}} = R_0/[1 \pm \frac{R_0}{2d} - (\frac{R_0}{2d})^4], \qquad [4]$$

with the bubble radius $R_0 = 85\,\mathrm{\mu m}$ and the distance $2\,d = 2\,R_0$ between the bubble centres. The signs introduced by $\pm$ are required to denote the location of each bubble centre such that normal velocities midway between the bubbles, *i.e.*, at the location of the rigid wall or glass slide, vanish.

The derivation of the capacitance now allows for the correction of the wall effect for a spherical bubble's natural resonance frequency in PDMS as follows:

$$f_{\mathrm{PDMS,W}} = 0.833 \cdot f_{\mathrm{PDMS,0}} \approx 32.2\,\mathrm{kHz}, \qquad [5]$$

where 0.833 has been obtained from M. Strasberg. The Pulsation Frequency of Nonspherical Gas Bubbles in Liquids. The Journal of the Acoustical Society of America (1953) **25**. (8) for the case where $d = R_0$ and $f_{\mathrm{PDMS,0}} = 38.6\,\mathrm{kHz}$ corresponds to the natural resonance frequency of a spherical microbubble in PDMS (see Supporting Text 3).

While the derived natural frequency decreases as expected if a rigid boundary, such as the glass slide, is introduced, it is important to highlight that this derivation relies on significant assumptions including but not limited to the previous the calculation of the natural frequency in a PDMS environment. Furthermore, it is worth noting that the resonance frequency $f_{\mathrm{PDMS,W}}$ does not account for the shape-anisotropy of the air chamber in the embedded microbubbles and, hence, an increase in frequency in the range of 10 % would be expected (based on Equation (11) and (12) provided in the manuscript). However, despite such an increase, the analytical model of a microbubble's natural frequency proves to be insufficient to account for the complex relation between the various components of the embedded microbubbles and, as such, is unable to describe the resonance frequencies and resulting manipulation capabilities observed in numerical as well as experimental investigations.

3. Supporting Videos

A. Video SV-1.

Supporting Information SV-1: Stable out-of-plane motion of 10 µm polystyrene particles. The particles are trapped *via* radiation forces in front of an embedded microbubble with a wall thickness $T = 10\,\mu m$ and follow the developed out-of-plane streaming. The excitation frequency and voltage are 68 kHz and $10\,V_{PP}$, respectively. Scale bar = 50 µm.

B. Video SV-2.

Supporting Information SV-2: Vibration of an embedded microbubble with a wall thickness $T = 5\,\mu m$ during a frequency sweep from $68 - 70$ kHz. The excitation voltage of $20\,V_{PP}$ is kept constant. The vibration is recorded at 40 000 frames/s. The video is played back at 16 frames/s which corresponds to a reduction by a factor of $2666.7x$. Scale bar = 25 µm.

C. Video SV-3.

Supporting Information SV-3: The vibration of an embedded microbubble with a wall thickness $T = 5\,\mu m$ when the excitation signal is switched from OFF to ON. The excitation frequency and voltage are 68.9 kHz and $20\,V_{PP}$, respectively. The location of maximum displacement is clearly visible near the centre of the PDMS wall. The vibration is recorded at 40 000 frames/s and the video is played back at 15 fps which corresponds to a reduction by a factor of $2666.7x$. Scale bar = 25 µm.

D. Video SV-4.

Supporting Information SV-4: Stable out-of-plane rotation of a single HeLa cell. The single cell is trapped in the pressure node near an embedded microbubble with a 5 µm thin wall and slowly rotated using the generated out-of-plane vortices. The embedded microbubble is excited at a frequency of 67 kHz with $6\,V_{PP}$. The video offers an additional view of the manipulated specimen with a focus on the PDMS wall. Scale bar = 25 µm.

E. Video SV-5.

Supporting Information SV-5: Mixing capabilities of the acoustic device. Black dye and de-ionised water are mixed using a series of embedded microbubbles. The microstructures are excited at a frequency of 69 kHz. The videos demonstrate the mixing for excitation voltages of $14\,V_{PP}$ and $20\,V_{PP}$, respectively. For demonstration purposes, the acoustic activation is switched on and off. Scale bar = 500 µm.

References

1. A Selfridge, Approximate material properties in isotropic materials. *IEEE Transactions on Sonics Ultrasonics* **32**, 381–394 (1985).
2. Massachusetts Institute of Technology, PDMS Material Properties (http://www.mit.edu/~6.777/matprops/pdms.htm) (2004) Accessed: 2020-12-06.
3. PB Muller, R Barnkob, MJH Jensen, H Bruus, A numerical study of microparticle acoustophoresis driven by acoustic radiation forces and streaming-induced drag forces. *Lab Chip* **12**, 4617–4627 (2012).
4. JT Karlsen, H Bruus, Forces acting on a small particle in an acoustical field in a thermoviscous fluid. *Phys. Rev. E* **92**, 043010 (2015).
5. SL Lai, D Johnson, R Westerman, Aspect ratio dependent etching lag reduction in deep silicon etch processes. *Journal Vacuum Science & Technology A: Vacuum, Surfaces, Films* **24**, 1283–1288 (2006).
6. M Minnaert, XVI.on musical air-bubbles and the sounds of running water. *The London, Edinburgh, Dublin Philosophical Magazine Journal Science* **16**, 235–248 (1933).
7. KS Spratt, MF Hamilton, KM Lee, PS Wilson, Radiation damping of, and scattering from, an arbitrarily shaped bubble. *The Journal Acoustical Society America* **142**, 160–166 (2017).
8. M Strasberg, The pulsation frequency of nonspherical gas bubbles in liquids. *The Journal Acoustical Society America* **25**, 536–537 (1953).
9. KS Spratt, KM Lee, PS Wilson, MS Wochner, On the resonance frequency of an ideal arbitrarily-shaped bubble in *Proceedings of Meetings on Acoustics.* (Acoustical Society of America), Vol. 20, p. 045004 (2014).
